# Inhibition of MNKs promotes macrophage immunosuppressive phenotype to limit CD8^+^ T cell antitumor immunity

**DOI:** 10.1172/jci.insight.152731

**Published:** 2022-05-09

**Authors:** Thao N.D. Pham, Christina Spaulding, Mario A. Shields, Anastasia E. Metropulos, Dhavan N. Shah, Mahmoud G. Khalafalla, Daniel R. Principe, David J. Bentrem, Hidayatullah G. Munshi

**Affiliations:** 1 Department of Medicine, Feinberg School of Medicine, Northwestern University, Chicago, Illinois, USA.; 2 Jesse Brown VA Medical Center, Chicago, Illinois, USA.; 3 The Robert H. Lurie Comprehensive Cancer Center, Chicago, Illinois, USA.; 4 Department of Surgery, Feinberg School of Medicine, Northwestern University, Chicago, Illinois, USA.; 5 Medical Scientist Training Program, College of Medicine, University of Illinois at Chicago, Chicago, Illinois, USA.

**Keywords:** Immunology, Immunotherapy, Macrophages, T cells

## Abstract

To elicit effective antitumor responses, CD8**^+^** T cells need to infiltrate tumors and sustain their effector function within the immunosuppressive tumor microenvironment (TME). Here, we evaluate the role of MNK activity in regulating CD8**^+^** T cell infiltration and antitumor activity in pancreatic and thyroid tumors. We first show that human pancreatic and thyroid tumors with increased MNK activity are associated with decreased infiltration by CD8**^+^** T cells. We then show that, while MNK inhibitors increase CD8**^+^** T cells in these tumors, they induce a T cell exhaustion phenotype in the tumor microenvironment. Mechanistically, we show that the exhaustion phenotype is not caused by upregulation of programmed cell death ligand 1 (PD-L1) but is caused by tumor-associated macrophages (TAMs) becoming more immunosuppressive following MNK inhibitor treatment. Reversal of CD8**^+^** T cell exhaustion by an anti–PD-1 antibody or TAM depletion synergizes with MNK inhibitors to control tumor growth and prolong animal survival. Importantly, we show in ex vivo human pancreatic tumor slice cultures that MNK inhibitors increase the expression of markers associated with immunosuppressive TAMs. Together, these findings demonstrate a role of MNKs modulating a protumoral phenotype in macrophages and identify combination regimens involving MNK inhibitors to enhance antitumor immune responses.

## Graphical abstract

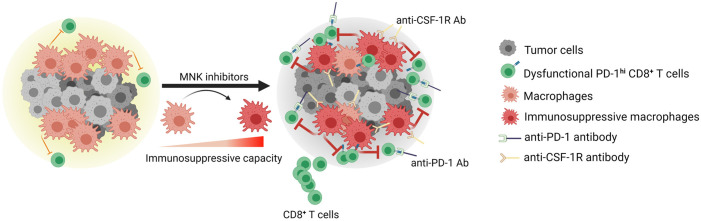

## Introduction

Programmed cell death 1/Programmed cell death ligand 1 (PD-1/PD-L1) checkpoint blockade presents a promising anticancer treatment modality (1, 2). However, single-agent therapy with either anti–PD-1 or PD-L1 antibodies has failed to elicit meaningful responses in many tumor types — e.g., glioblastoma (3), pancreatic ductal adenocarcinoma (PDAC) (4, 5), and differentiated thyroid cancers (6). Seminal studies identify tumor-infiltrating CD8**^+^** T lymphocytes as a prime predictor of responses to T cell–based immunotherapies (7, 8). As a result, identifying molecular mechanisms regulating CD8**^+^** T cell infiltration and function will likely broaden the therapeutic scope of immune checkpoint therapies.

Macrophages account for one of the most abundant immune cell types within the tumor microenvironment (TME) (9, 10). In general, macrophages can be classified as either classically activated (M1) macrophages or alternatively activated (M2) macrophages (9, 10). While M1 macrophages can produce proinflammatory cytokines and initiate an immune response against tumor cells, M2 macrophages and TAMs tend to exert an immunosuppressive phenotype, favoring tumor progression (9, 10). Previously, it was demonstrated that physical engagement between the incoming CD8**^+^** T cells and TAMs reduces the motility of T cells in the stroma, limiting their entry into tumor nests (11). TAMs can also inhibit CD8**^+^** T cell function by expressing immune checkpoint ligands (e.g., PD-L1) (12, 13), secreting immunosuppressive cytokines (e.g., TGF-β, LIF, CCL22) (9, 10), and limiting metabolites required for T cell proliferation (e.g., L-arginine by expressing Arginase-1 enzyme) (14–16). Efforts to inhibit or deplete TAMs have demonstrated promising antitumor efficacy in several preclinical models by increasing CD8**^+^** T cell infiltration and reducing local immunosuppressive signals (11, 17). Additionally, TAMs can limit the efficacy of anti–PD-1/PD-L1 therapy (18, 19), as targeting TAMs synergizes with anti–PD-1/PD-L1 antibodies to reactivate CD8**^+^** T cells and achieve more potent tumor control (11, 17). These findings highlight TAMs as promising immunomodulatory targets and provide the rationale for investigating additional signaling pathways that govern macrophage polarization.

MAPK-interacting serine/threonine-protein kinase 1 and 2 (MNK1 and MNK2, respectively) are phosphorylated and activated by the MEK/ERK and the p38 MAPK pathways (20, 21). Activation of MNKs allows them to phosphorylate and activate their substrates, most notably eukaryotic initiation factor 4E (eIF4E) (22). Increased expression and activity of MNKs can promote tumor growth and therapeutic resistance, as demonstrated by several independent studies (23–28). More recently, the immunomodulatory role of MNKs has become a subject of active investigation. Pharmacological inhibition of MNKs in a mouse model of metastatic hepatocellular carcinoma reduced cancer cell–specific PD-L1 and restored immune surveillance (29). However, in other mouse models, it has been reported that MNKs modulate the function of immune cells to drive local immunosuppression. For instance, in the MMTV-PyMT mouse model of breast cancer, MNK2 is required for the antiinflammatory phenotype in TAMs (30). In a mouse model of melanoma, both MNKs appear to promote an immunosuppressive phenotype in DCs (31). These studies demonstrate the previously underrecognized immunomodulatory roles of MNKs and suggest that the effects of MNK inhibition on the tumor immune microenvironment vary depending on the tumor type.

In this study, we explored the effects of MNK inhibitors in mouse PDAC and papillary thyroid tumors. Previously, we showed a tumor-promoting role of MNKs in PDAC and thyroid cancer cells (26, 32). Here, we evaluated the effects of MNK inhibitors on CD8**^+^** T cell infiltration and function in these tumors. Initially, we showed that human PDAC and differentiated thyroid tumors with increased MNK activity are associated with decreased infiltration by CD8**^+^** T cells. We then showed that, while MNK inhibitors increased CD8**^+^** T cells in tumors, MNK inhibitors induced a T cell exhaustion phenotype in the TME. The exhaustion phenotype is not caused by upregulation of PD-L1 but is caused by TAMs becoming more immunosuppressive following MNK inhibitor treatment. Importantly, reversal of CD8**^+^** T cell exhaustion by an anti–PD-1 antibody or TAM depletion synergizes with MNK inhibitors to control tumor growth and prolong animal survival. To support the findings in our animal studies, we showed in ex vivo human PDAC slice cultures that MNK inhibitors increased the expression of markers associated with immunosuppressive TAMs. Together, these findings demonstrated a previously unknown role of MNKs in modulating a protumoral phenotype in macrophages and identified combination regimens involving MNK inhibitors to enhance antitumor immune responses.

## Results

### Increased MNK activity correlates with decreased CD8^+^ T cell infiltration in human tumors.

**Figure 1:**
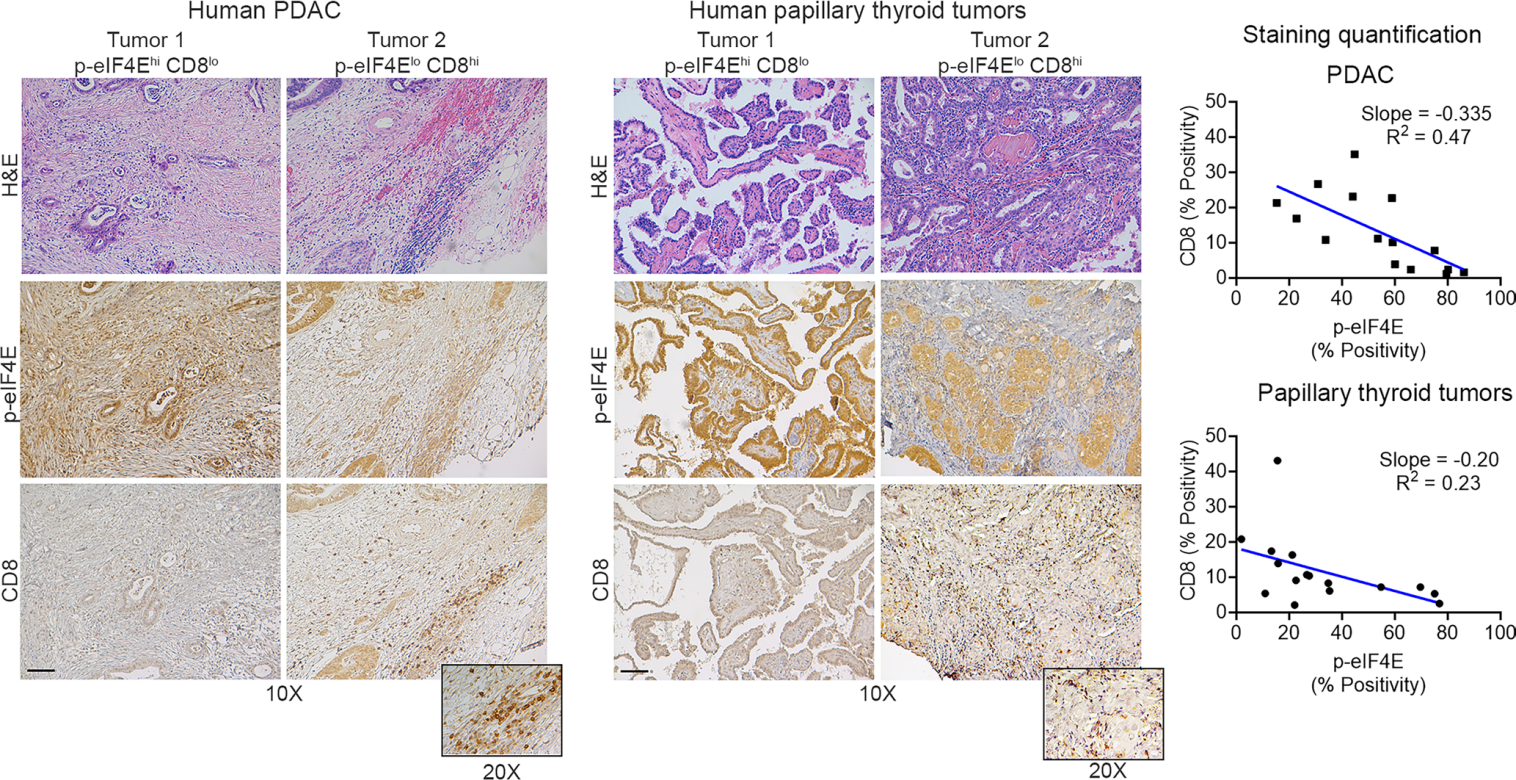
Increased MNK activity correlates with decreased CD8^+^ T cell infiltration in human tumors. Serial sections of deidentified human pancreatic ductal adenocarcinoma (PDAC) (*n* = 15) and papillary thyroid tumors (*n* = 16) were H&E stained and stained by IHC for CD8**^+^** T cells and eIF4E**^S209^** phosphorylation (p-eIF4E). Staining positivity was captured by the TissueGnostics Imaging system and analyzed by HistoQuest. Correlation analysis for CD8**^+^** T cells and p-eIF4E expression was performed using GraphPad. Scale bar: 200 μm.

MNKs are the sole kinases of eIF4E, as targeting of MNKs (MNK1 and MNK2) abrogates both basal and stimuli-induced phosphorylation of eIF4E (33). Using expression levels of phosphorylated eIF4E (p-eIF4E**^S209^**) as the readout for MNK activity, we evaluated the relationship between MNK activity and CD8**^+^** T cells. We found that human PDAC and papillary thyroid tumors with increased p-eIF4E**^S209^** are associated with decreased infiltration by CD8**^+^** T cells (Figure 1).

### MNK inhibitors increase CD8^+^ T cells in tumors but induce a T cell exhaustion phenotype in the tumor microenvironment.

**Figure 2:**
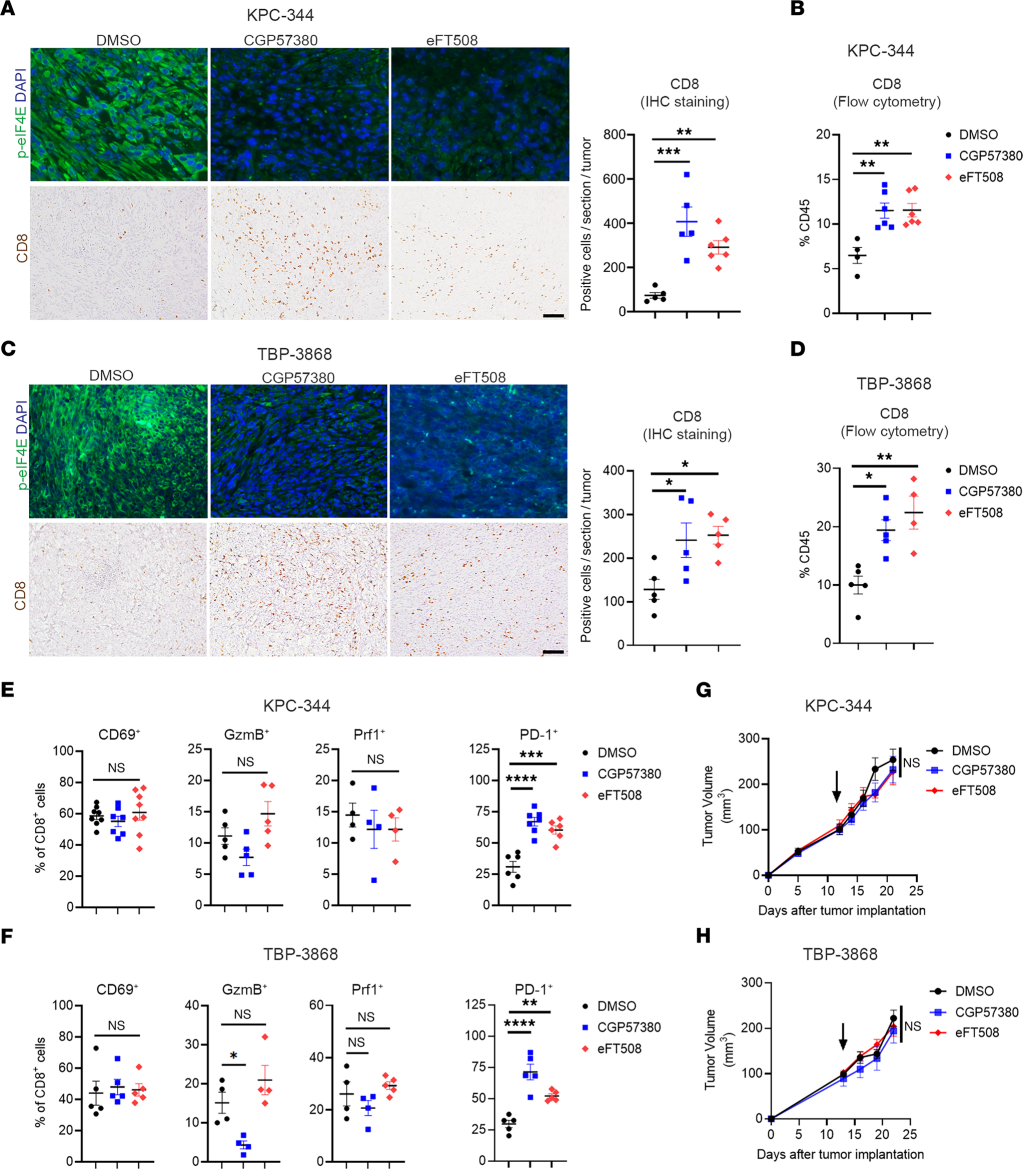
MNK inhibitors increase CD8^+^ T cells in tumors but induce a T cell exhaustive phenotype. Mice with established syngeneic pancreatic (KPC-344) and thyroid (TBP-3868) tumors were randomized and treated with DMSO (vehicle control), the MNK inhibitor CGP57380 (25 mg/kg), or the MNK inhibitor eFT508 (1 mg/kg) daily. (**A** and **C**) The collected tumors were analyzed for p-eIF4E expression by immunofluorescence staining and CD8**^+^** T cells by IHC staining. Scale bar: 100 μm. The absolute number of stained CD8**^+^** T cells per 10× section was quantified by ImageJ and averaged from 5 individual sections. (**B** and **D**) The collected tumors were digested and analyzed by flow cytometry for the number of CD8**^+^** T cells. (**E** and **F**) Tumor-infiltrating CD8**^+^** T cells were analyzed by flow cytometry for the expression of CD69, granzyme B (GzmB), perforin-1 (Prf1), and PD-1. Data points in **B**, **D**, **E**, and **F** represent individual tumors. (**G** and **H**) Tumor size was monitored and measured using calipers. Tumor volumes (V) were calculated using the formula *V* = (*W**^2^*** × *L*)/2, where *W* is tumor width and *L* is tumor length. Arrows indicate start of treatment. Data are shown as the mean ± SEM, and analysis was done using 1-way ANOVA followed by Dunnett’s multiple comparison test. *P < 0.05; **P < 0.01; ***P < 0.001; ****P < 0.0001.

We next evaluated the effects of targeting MNKs on CD8**^+^** T cell infiltration and activity. We treated mouse pancreatic and thyroid tumors with 2 known MNK inhibitors, CGP57380 (34) and eFT508 (Tomivosertib) (35), which effectively decreased p-eIF4E**^S209^** levels in vivo (Figure 2, A and C). MNK inhibitors significantly enhanced CD8^+^ T cell infiltration, measured by IHC staining and flow cytometry (Figure 2, A–D). However, they did not affect the presence of other immune cell populations, specifically CD4**^+^** T cells, Tregs, polymorphonuclear myeloid–derived suppressor cells (P-MDSCs), NK cells, or B cells (Supplemental Figure 1; supplemental material available online with this article; https://doi.org/10.1172/jci.insight.152731DS1). There was also increased expression of known T cell chemoattractants in tumors treated with MNK inhibitors (Supplemental Figure 2). However, the CD8^+^ T cells in the tumors did not express markers for T cell activation and cytolytic activity, such as CD69, granzyme B (GzmB), or perforin-1 (Prf1) (Figure 2, E and F). Instead, MNK inhibitors increased PD-1 expression in tumor-infiltrating CD8^+^ T cells in both the KPC-344 and the TBP-3868 tumors (Figure 2, E and F, and Supplemental Figure 3A). In contrast, MNK inhibitors did not affect the expression of PD-1, Prf1, or CD69 in CD8^+^ T cells isolated from the spleens of tumor-bearing mice treated with MNK inhibitors in vivo (Supplemental Figure 3B). Also, MNK inhibitors did not affect the expression of PD-1, Prf1, or CD69 in CD8**^+^** T cells isolated from the spleens of non–tumor-bearing mice and treated with MNK inhibitors ex vivo (Supplemental Figure 3C). In addition, while MNK inhibitors did not affect LAG3 expression in tumor-infiltrating CD8**^+^** T cells in both the KPC-344 and the TBP-3868 tumors, MNK inhibitors increased TIM3 levels in tumor-infiltrating CD8**^+^** T cells only in the KPC-344 tumors (Supplemental Figure 4). There was also increased coexpression of PD-1 and TIM3 on tumor-infiltrating CD8^+^ T cells in the KPC-344 tumors (Supplemental Figure 4). Overall, these results suggest that, while MNK inhibitors increased CD8^+^ T cells in tumors, MNK inhibitors induced a T cell exhaustion phenotype in the tumor microenvironment. Consistent with these findings, MNK inhibitors did not inhibit tumor growth (Figure 2, G and H), nor did they affect tumor proliferation or apoptosis (Supplemental Figure 5).

### Anti–PD-1 antibody synergizes with MNK inhibitors in vivo.

**Figure 3:**
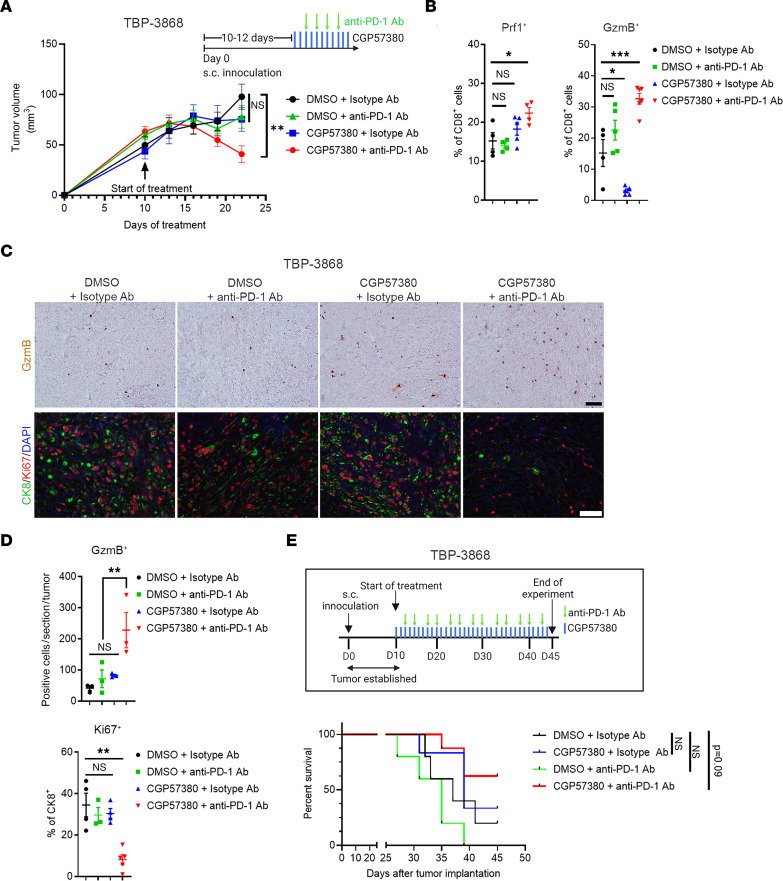
Anti–PD-1 antibody synergizes with MNK inhibitors. Mice with established TBP-3868 tumors were randomized and treated with CGP57380 (25 mg/kg, daily) or vehicle control (DMSO) in combination with an anti–PD-1 antibody (200 μg, twice weekly) or a control isotype-matched IgG antibody (200 μg, twice weekly). (**A**) Tumor size was measured daily by caliper, and tumor volume was calculated using the formula *V* = (*W**^2^*** × *L*)/2. (**B**) At the study endpoint, the tumor-infiltrating CD8**^+^** T cells were isolated and analyzed by flow cytometry for perforin-1 (Prf1) and granzyme B (GzmB). (**C** and **D**) Tumors collected at the endpoint were stained by IHC for GzmB (top panel) and were immunofluorescence costained for cytokeratin 8 (CK8) and Ki67 and counterstained with DAPI (bottom panel). Scale bar: 50 μm. The absolute number of GzmB**^+^** cells per 10× section and the number of Ki67**^+^** cells as a percentage of CK8**^+^** cells were quantified by ImageJ and analyzed by GraphPad. Data points in **B** and **D** represent individual tumors. (**E**) The effect of different treatments on the overall survival of TBP-3868 tumor-bearing mice was determined as described in Methods. Log-ranked (Mantel-Cox) test for survival analysis was performed by GraphPad. In **B** and **D,** data are shown as the mean ± SEM, and analysis was done using 1-way ANOVA followed by Dunnett’s multiple comparison test. *P < 0.05; **P < 0.01; ***P < 0.001.

**Figure 4:**
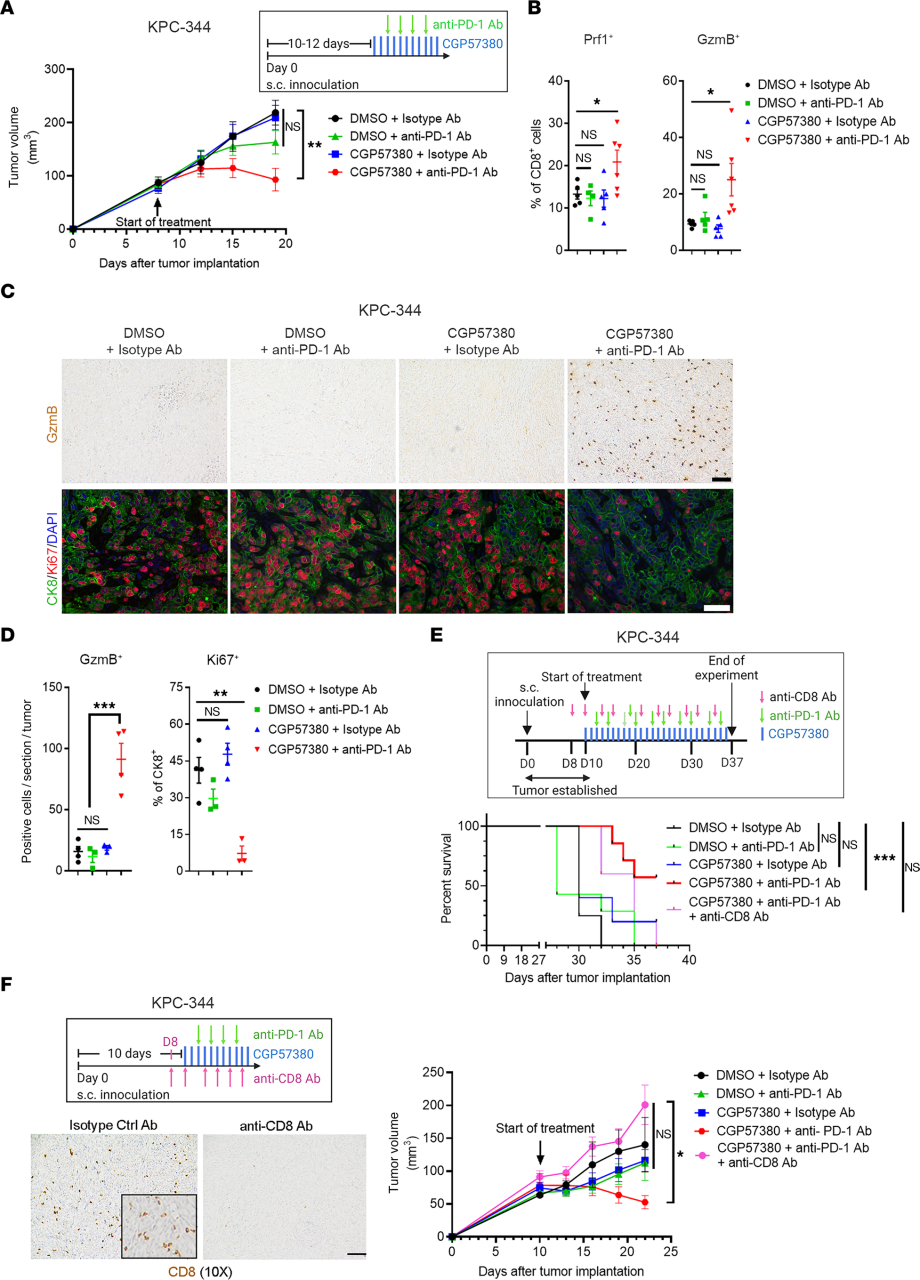
Antitumor efficacy of combined anti–PD-1 antibody and MNK inhibitors is dependent on CD8^+^ T cells. Mice with KPC-344 tumors were randomized and treated with CGP57380 (25 mg/kg, daily) or DMSO in combination with an anti–PD-1 antibody (200 μg, twice weekly) or an isotype-matched IgG antibody (200 μg, twice weekly). (**A**) Tumor size was measured daily, and tumor volume was calculated using the formula *V* = (*W***^2^** × *L*)/2. (**B**) At the study endpoint, the tumor-infiltrating CD8**^+^** T cells were isolated and analyzed by flow cytometry for perforin-1 (Prf1) and granzyme B (GzmB). (**C** and **D**) Tumors collected at the endpoint were stained by IHC for GzmB (top panel) and were immunofluorescence costained for cytokeratin 8 (CK8) and Ki67 and counterstained with DAPI (bottom panel). Scale bar: 50 μm. The absolute number of GzmB**^+^** cells per 10× section and the number of Ki67**^+^** cells as a percentage of CK8**^+^** cells were quantified by ImageJ and analyzed by GraphPad. Data points in **B** and **D** represent individual tumors. (**E**) In addition to treating with CGP57380 and an anti–PD-1 antibody, KPC-344 tumor-bearing mice were cotreated with a CD8**^+^** T cell–depleting antibody (300 μg, on day –2, day 0, and then twice weekly). The overall survival of KPC-344 tumor-bearing mice was determined as described in Methods. Log-ranked (Mantel-Cox) test for survival analysis was performed by GraphPad. ***P < 0.001. (**F**) The KPC-344 tumor-bearing mice were treated with CGP57380 and an anti–PD-1 antibody and cotreated with a CD8**^+^** T cell–depleting antibody. Tumor volume was calculated as described above. The efficacy of the anti-CD8 antibody was confirmed by IHC staining for CD8. In **B**, **D**, and **F,** data are shown as the mean ± SEM, and analysis was done using 1-way ANOVA followed by Dunnett’s multiple comparison test. *P < 0.05; **P < 0.01; ***P < 0.001.

Given the increased PD-1 levels on tumor-infiltrating CD8**^+^** T cells following MNK inhibitor treatment, we evaluated whether cotreatment with an anti–PD-1 antibody could overcome T cell exhaustion and suppress tumor growth. We treated mouse thyroid and pancreatic tumors with CGP57380 or DMSO (vehicle control) in combination with either an anti–PD-1 antibody or a control isotype-matched IgG antibody. While single-agent treatment with CGP57380 or an anti–PD-1 antibody did not affect tumor growth, the combination therapy significantly inhibited tumor growth (Figure 3A and Figure 4A). Mechanistically, we found that the combination therapy increased the number of Prf1- and GzmB-expressing CD8^+^ T cells, increased tumoral GzmB expression, and decreased tumor cell proliferation (Figure 3, B–D, and Figure 4, B–D). Similarly, the anti–PD-1 antibody synergized with eFT508, a MNK inhibitor currently in clinical trials (36), at controlling tumor growth in vivo (Supplemental Figure 6). Notably, the combination therapy prolonged the overall survival of tumor-bearing mice (Figure 3E and Figure 4E). Importantly, cotreatment with an anti-CD8 antibody abrogated the efficacy of the combination treatment (Figure 4, E and F), demonstrating the requirement of CD8**^+^** T cells in mediating the response to the combination treatment.

### Targeting MNKs does not affect PD-L1 expression.

**Figure 5:**
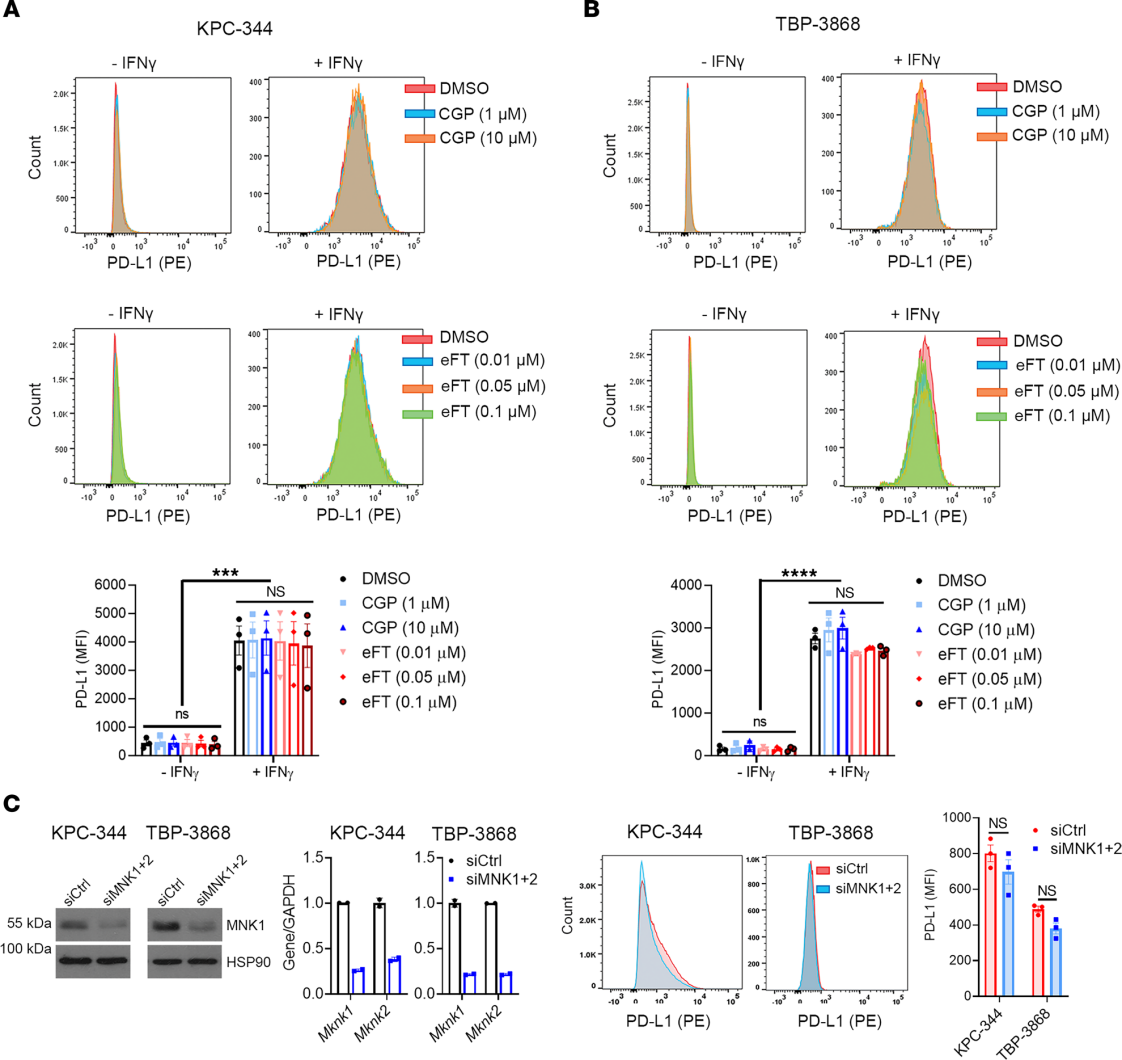
Targeting MNKs does not affect PD-L1 expression. (**A** and **B**) Mouse pancreatic (KPC-344) and thyroid (TBP-3868) cancer cells were pretreated with DMSO, CGP57380 (CGP; 1, 10 μM), or eFT508 (eFT; 0.01, 0.05, 0.1 μM) for 24 hours and then treated with either vehicle control or IFN-γ (200 ng/mL) for additional 24 hours. The cells were then analyzed for PD-L1 expression by flow cytometry, and the mean fluorescence intensity (MFI) was calculated using FlowJo. (**C**) Mouse cancer cells were transfected with siRNAs targeting *Mknk1* and *Mknk2* (siMNK1+2) for 48 hours. Knockdown efficiency was confirmed by Western blotting for MNK1 and qPCR for *Mknk1* and *Mknk2* mRNAs. Data are shown as the mean ± SD from 2 technical replicates. Data are representative of 3 independent biological replicates. The cells were then analyzed for PD-L1 by flow cytometry, and the MFI was calculated using FlowJo. In **C**, data are shown as the mean ± SEM from 3 biological replicates. Unpaired *t* test. In **A** and **B**, data are shown as the mean ± SEM from 3 biological replicates, and analysis was performed by 2-way ANOVA; ***P < 0.001; ****P < 0.0001.

To understand the mechanism for T cell exhaustion following MNK inhibitor treatment, we first evaluated whether MNK inhibitors affected cell surface PD-L1 expression. While treatment with eFT508 suppresses PD-L1 expression in cell lines derived from a Myc-driven hepatocellular carcinoma transgenic mouse model (29), we found that neither CGP57380 nor eFT508 affected PD-L1 expression levels in mouse pancreatic and thyroid cancer cells (Figure 5, A and B). We validated these findings by flow cytometry with a second anti–PD-L1 antibody (Supplemental Figure 7, A–D) and by Western blotting (Supplemental Figure 7, E and F). Similarly, treatment with eFT508 did not affect cell surface PD-L1 expression in human pancreatic and thyroid cancer cells (Supplemental Figure 7G). Additionally, MNK1/2 knockdown using siRNA did not affect cell surface PD-L1 levels (Figure 5C). Finally, we found no difference in PD-L1 expression on TAMs, myeloid cells, and Tregs isolated from the control and MNK inhibitor–treated mice (Supplemental Figure 8, A and B). Consistent with these findings, there was no change in whole tumor expression of PD-L1 with MNK inhibitor treatment (Supplemental Figure 8, C and D).

### Inhibiting MNKs enhances an immunosuppressive phenotype in macrophages.

**Figure 6:**
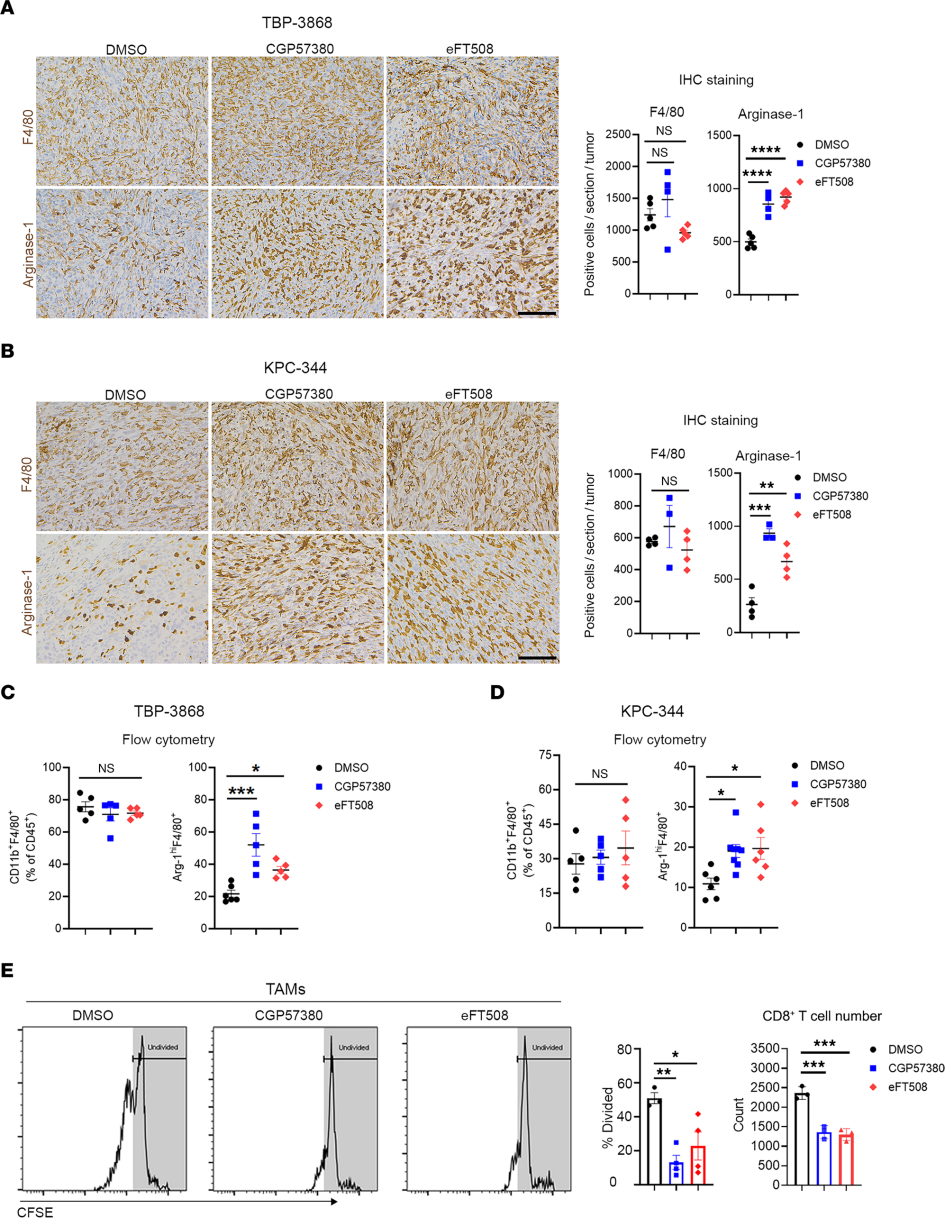
Inhibiting MNKs enhances an immunosuppressive phenotype in tumor-associated macrophages (TAMs). Established syngeneic mouse thyroid (TBP-3868) and pancreatic (KPC-344) tumors were treated with CGP57380 (25 mg/kg) or eFT508 (1 mg/kg) daily for 2 weeks. (**A** and **B**) The tumors were stained for F4/80 and Arginase-1 by IHC. Scale bar: 100 μm. The absolute number of F4/80^+^ and Arginase-1^+^ cells per 20× field was quantified by ImageJ and analyzed by GraphPad. (**C** and **D**) The effects of MNK inhibitors on the number of TAMs (CD11b**^+^**F4/80**^+^**) and their expression of Arginase-1 (Arg-1^hi^F4/80**^+^**) were analyzed by flow cytometry. (**E**) TAMs (CD45**^+^**CD11b**^+^**F4/80**^+^**) collected from mice treated with DMSO, CGP57380, or eFT508 were cocultured with CFSE-stained splenic CD8**^+^** T cells for 96 hours. T cell proliferation, as determined by the percentages of dividing T cells and T cell numbers, was analyzed by flow cytometry. Data are shown as the mean ± SEM. Data points in **A**–**D** represent individual tumors, and data points in **E** represent biological replicates. Analysis was done using 1-way ANOVA followed by Dunnett’s multiple comparison test. *P < 0.05; **P < 0.01; ***P < 0.001; ****P < 0.0001.

Tumor-associated macrophages (TAMs) are among the most abundant immune cell populations in the pancreatic TME (9, 10). In addition to suppressing CD8**^+^** T cells through PD-L1/PD-1 interaction (12, 13), TAMs can express immunosuppressive cytokines and enzymes, including Arginase-1 (14–16). Thus, we evaluated the effects of MNK inhibitors on TAMs. While MNK inhibitors did not alter the number of TAMs, we found that MNK inhibitors significantly increased Arginase-1 expression in TAMs (Figure 6, A–D). Moreover, TAMs isolated from MNK inhibitor–treated tumors were more potent at suppressing T cell proliferation ex vivo (Figure 6E).

**Figure 7:**
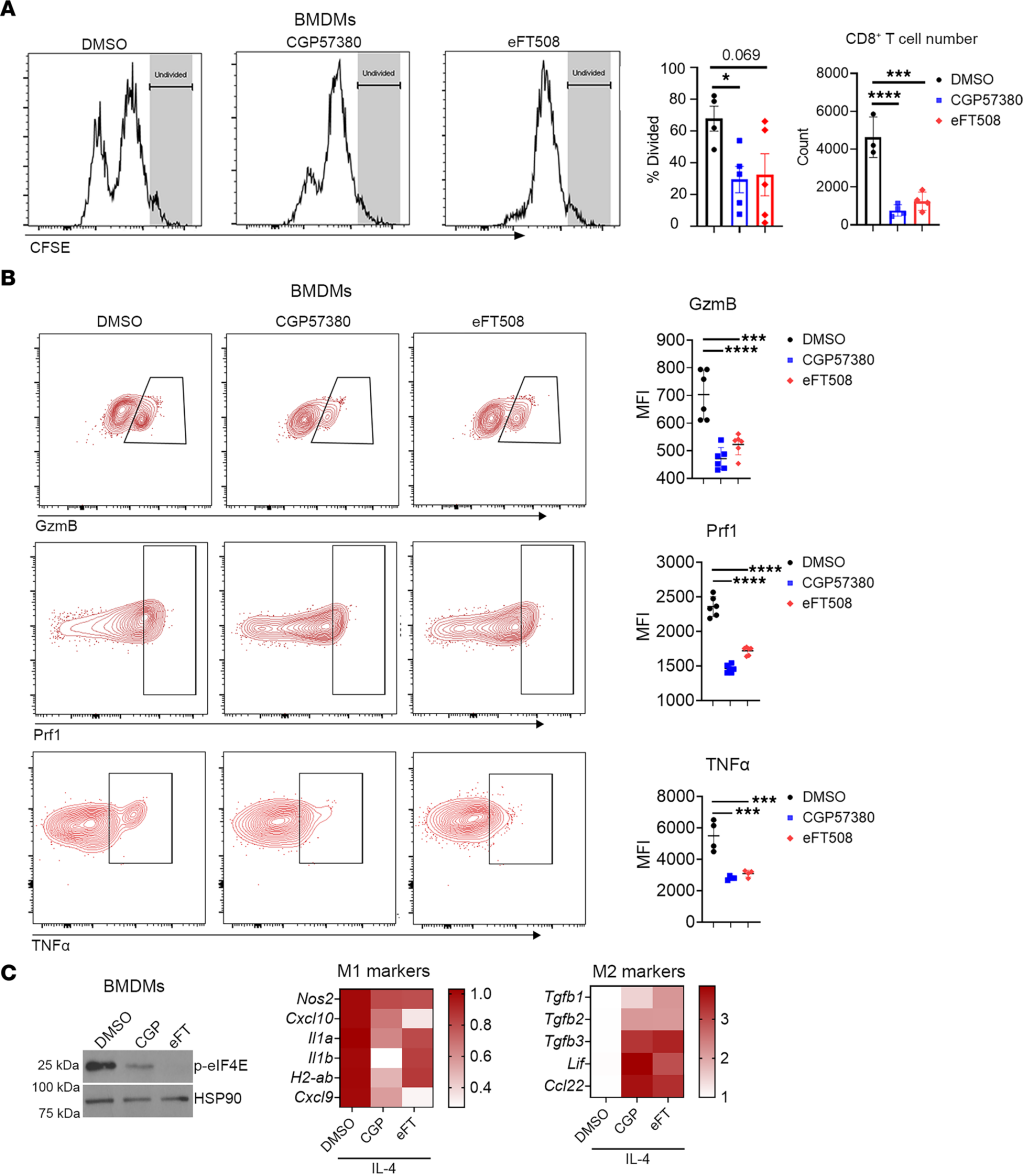
MNK inhibitors potentiate an immunosuppressive phenotype in BM-derived monocytes (BMDMs). (**A**) BMDMs were cultured in L929-conditioned media for 5 days before treating for 24 hours with IL-4 (25 ng/mL) to induce alternatively activated (M2) polarization. M2-polarized BMDMs were cocultured with CFSE-stained splenic CD8**^+^** T cells for 72 hours in the presence of DMSO, CGP57380 (10 μM), or eFT508 (1 μM). T cell proliferation, as determined by the percentages of dividing T cells and T cell numbers, was analyzed by flow cytometry. (**B**) The CD8**^+^** T cells were also analyzed by flow cytometry for the expression of granzyme B (GzmB), perforin-1 (Prf1), and TNF-α. MFI was calculated using FlowJo. (**C**) M2-polarized BMDMs were treated with DMSO, CGP57380 (CGP, 10 μM), or eFT508 (eFT, 1 μM) for 48 hours. Expression of phosphorylated eIF4E^S209^ (p-eIF4E) was analyzed by Western blotting with HSP90 as loading control. Expression levels of select classically activated (M1) and M2 genes were evaluated by qPCR with GAPDH used as a house-keeping gene and averaged from 3 independent experiments to generate heatmaps. Data and data points in **A** are shown as the mean ± SEM and biological replicates respectively. Data and data points in **B** are shown as the mean ± SD and technical replicates, respectively, and data are representative of 3 independent experiments. Analysis was done using 1-way ANOVA followed by Dunnett’s multiple comparison test. *P < 0.05; ***P < 0.001; ****P < 0.0001.

To better understand the effects of MNK inhibitors on macrophages, we isolated bone marrow-derived monocytes (BMDMs), polarized them to an immunosuppressive M2 phenotype in vitro, and cotreated them with MNK inhibitors. Compared with the control M2 BMDMs, the MNK inhibitor–treated M2 BMDMs were more effective at suppressing CD8**^+^** T cell proliferation (Figure 7A). In addition, CD8^+^ T cells cocultured with the MNK inhibitor–treated M2 BMDMs expressed lower levels of GzmB, Prf1, and TNF-α (Figure 7B). Consistent with an immunosuppressive phenotype, the MNK inhibitor–treated M2 BMDMs upregulated the expression of several known M2 genes (Figure 7C). Collectively, these data suggest MNK inhibitors enhance the ability of TAMs and M2 macrophages to suppress CD8**^+^** T cell effector function.

### Macrophage depletion in combination with MNK inhibitors reactivates CD8^+^ T cells and suppresses tumor growth in vivo.

**Figure 8:**
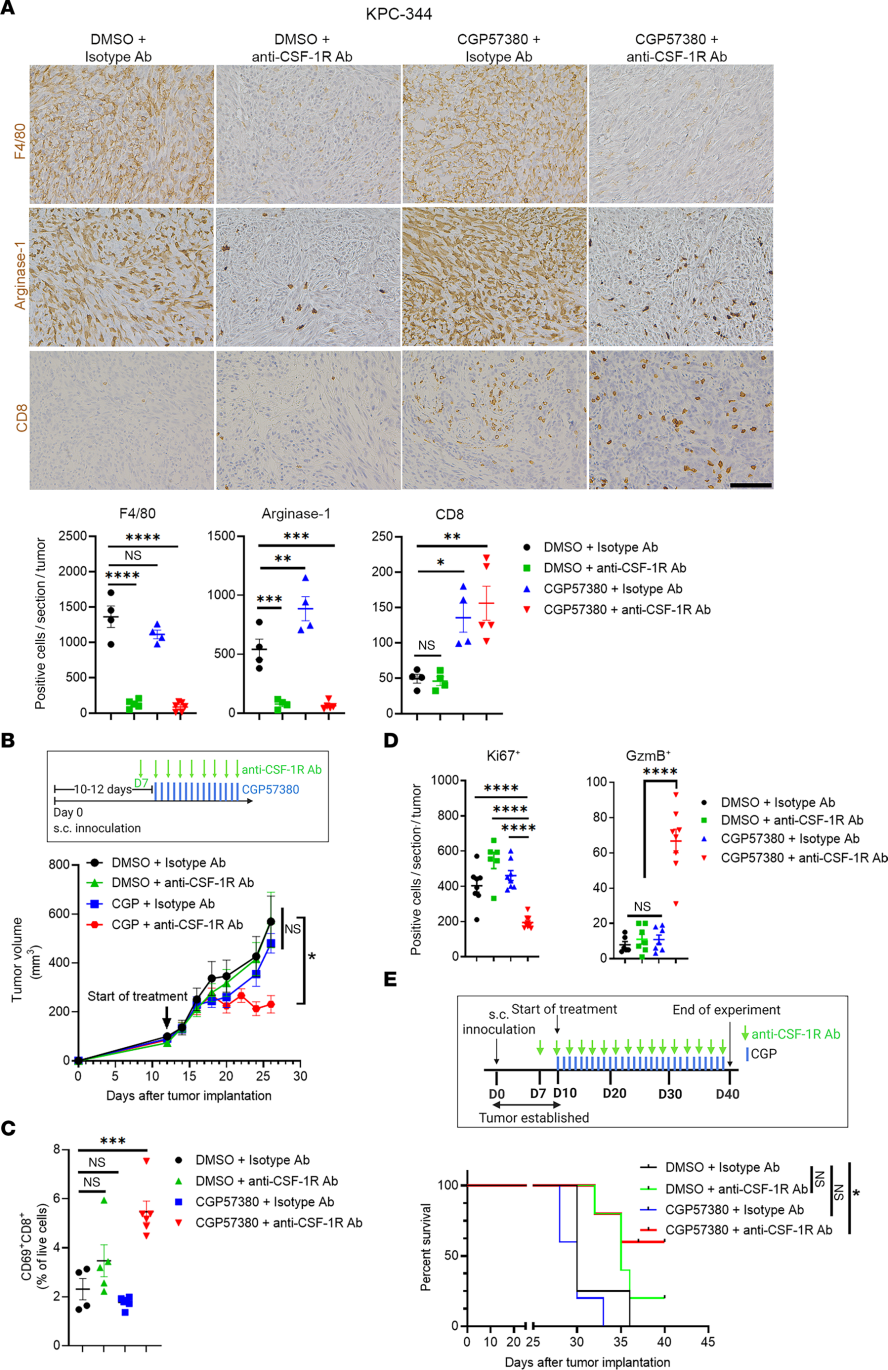
Macrophage depletion in combination with MNK inhibitors activates CD8^+^ T cells and suppresses tumor growth in vivo. Established KPC-344 tumors were treated with CGP57380 (CGP, 25 mg/kg, daily) or DMSO (vehicle control) in combination with either an anti–CSF-1R antibody (300 μg, day –3, day 0, and subsequently in an every-other-day pattern) or a control isotype-matched IgG antibody. (**A**) The tumors at the study endpoint were stained for F4/80, Arginase-1, and CD8 by IHC. Scale bar: 100 μm. The absolute number of positive cells per 20× field was quantified by ImageJ and analyzed by GraphPad. (**B** and **C**) KPC-344 tumor volume was measured by caliper and calculated using the formula *V* = (*W**^2^*** × *L*)/2. Isolated CD8**^+^** T cells from tumors collected at the study endpoint were analyzed for CD69 expression by flow cytometry. (**D**) Tumors were stained by IHC for Ki67 and granzyme B (GzmB). The number of positive cells per 10× field was quantified by ImageJ and analyzed by GraphPad. Data points in **A**–**D** represent individual tumors. (**E**) The effect of different treatments on the overall survival of tumor-bearing mice was determined as described in Methods. Log-ranked (Mantel-Cox) test for survival analysis was performed by GraphPad. Data in **A**–**D** are shown as the mean ± SEM, and analysis was done using 1-way ANOVA followed by Dunnett’s multiple comparison test. *P < 0.05; **P < 0.01; ***P < 0.001; ****P < 0.0001.

We next evaluated if depleting TAMs with an anti–CSF-1R antibody could synergize with MNK inhibitors to control tumor growth. In addition to depleting F4/80**^+^** TAMs, the anti–CSF-1R antibody significantly reduced Arginase-1 expression in tumors (Figure 8A). While the anti–CSF-1R antibody did not affect basal or MNK inhibitor–induced CD8^+^ T cell infiltration (Figure 8A), the anti–CSF-1R antibody synergized with CGP57380 to control tumor growth (Figure 8B). The combination treatment enhanced the expression of CD69 in the tumor-infiltrating CD8^+^ T cells (Figure 8C), increased tumoral GzmB (Figure 8D), and decreased tumor cell proliferation (Figure 8D). Similar to the combination treatment of CGP57380 and an anti–PD-1 antibody (Figures 3 and 4), the combination of CGP57380 and anti–CSF-1R antibody significantly prolonged the overall survival of tumor-bearing mice (Figure 8E). Together, these data suggest that TAMs play a major role in inhibiting cytolytic function of tumor-infiltrating CD8**^+^** T cells following treatment with MNK inhibitors.

### MNK inhibitors increase M2 markers in TAMs present in ex vivo human PDAC slice cultures.

**Figure 9:**
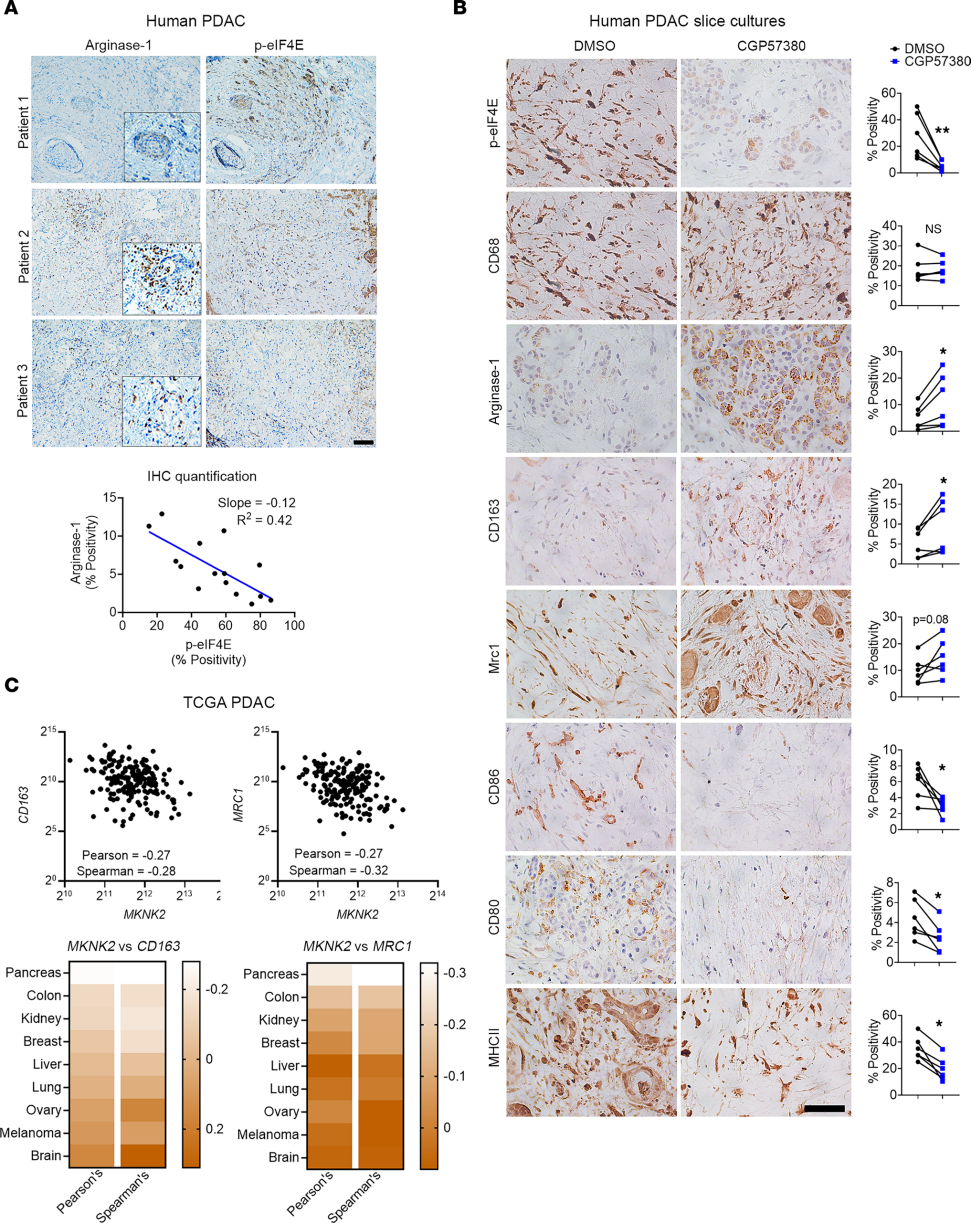
MNK inhibitors induce polarization of TAMs toward an M2 phenotype in ex vivo human PDAC slice cultures. (**A**) Serial sections of human PDAC tumors (*n* = 15) were stained for Arginase-1 and phosphorylated eIF4E**^S209^** (p-eIF4E) by IHC. Scale bar: 200 μm. The staining positivity was analyzed by the TissueGnostics Imaging system using the HistoQuest, and the correlation analysis for Arginase-1 and p-eIF4E expression was performed using GraphPad. (**B**) Slice cultures (*n* = 1–4 replicates for each treatment group) from 6 different PDAC tumors were treated with DMSO (vehicle control) or CGP57380 for 5 days and stained for phosphorylated eIF4E (p-eIF4E), CD68, Arginase-1, CD163, MRC1, CD86, CD80, and MHCII. The percentage of positive cells, relative to the total number of nucleated cells, was analyzed by ImageJ. Scale bar: 50 μm. Data points represent individual patients. Paired *t* test was performed by GraphPad. *P < 0.05; **P < 0.01. (**C**) The relative expression of *MKNK2*, *CD163*, and *MRC1* in TCGA studies and their transcript abundance from RNA-Seq data, quantified as RSEM, were downloaded from cBioPortal. Correlation analysis was performed in GraphPad to evaluate the relationship between *MKNK2*, *CD163*, and *MRC1*.

We initially evaluated the relationship between MNK activity and Arginase-1 expression in human PDAC tumors. Human PDAC tumors with low p-eIF4E**^S209^** levels were associated with high Arginase-1 expression (Figure 9A).

We also established ex vivo slice cultures of human PDAC tumors to further evaluate the effects of MNK inhibitors on TAMs. The slice cultures retain tissue integrity for up to 7 days and maintain the pronounced desmoplastic reaction present in human PDAC tumors (37, 38). While CGP57830 effectively reduced p-eIF4E**^S209^** levels (Figure 9B), it did not affect the number of TAMs, as demonstrated by CD68 staining. However, as seen in the animal studies (Figure 6), we found increased Arginase-1 expression in the CGP57830-treated slice cultures (Figure 9B). Treatment with CGP57380 also increased the expression of M2 macrophage markers CD163 and MRC1 (CD206) and decreased the expression of M1-associated, costimulatory markers CD86, CD80, and MHCII (Figure 9B). These data demonstrate that MNK inhibitors induced polarization of TAMs toward an M2 phenotype in ex vivo human PDAC slice cultures.

Finally, we interrogated the TCGA database to provide additional support for our findings that MNKs regulate the polarization of TAMs in human PDAC tumors. Compared with several other tumor types (Supplemental Figure 9), we found an inverse relationship between *MKNK2* and the M2 markers *CD163* and *MRC1* (*CD206*) in human PDAC tumors (Figure 9C). These data, together with our findings in animal studies, demonstrated a previously unknown role of MNKs in modulating a protumoral phenotype in macrophages.

## Discussion

Previously, it was found that increased expression and activity of MNKs promote resistance to chemotherapy and targeted therapies (23–28). In this work, we provide data showing that increased MNK activity also contributed to resistance to single-agent immune checkpoint inhibitors. We showed that high MNK activity is associated with low infiltration of CD8**^+^** T cells in human pancreatic and thyroid tumors. We also showed that pharmacological targeting of MNKs with small-molecule inhibitors increased tumor-infiltrating CD8**^+^** T cells in syngeneic tumor models. Notably, we demonstrated that inhibition of MNKs in TAMs promoted a T cell–suppressive M2 phenotype, resulting in T cell exhaustion. Moreover, MNK inhibitors enhanced the ability of both IL-4–polarized BMDMs and TAMs to suppress T cell proliferation ex vivo. Importantly, we validated our findings in animal studies using slice cultures established from resected human PDAC tumor specimens (37).

However, our findings contrast with the study by Bartish et al., in which they showed that targeting MNKs in breast cancer, particularly Mnk2, supports an antitumor phenotype in TAMs and enhances T cell function (30). Their findings suggest that single-agent treatment with MNK inhibitors is likely to produce effective T cell–mediated antitumor immunity. Since we found no evidence of CD8**^+^** T cell activation and tumor control following single-agent treatment with MNK inhibitors, our results suggest that targeting MNKs in TAMs may have differing effects depending on the tumor type. Notably, while we were able to identify a negative correlation between *MKNK2* and TAM M2 markers (*CD163*, *MRC1*) in the TCGA PDAC data set, this correlation was absent in the TCGA breast carcinoma data set, further highlighting the differences in tumor tissues likely accounting for the observed difference in TAM phenotype following MNK inhibition in pancreatic tumors compared with breast tumors.

Another study shows that SEL201, an ATP-competitive inhibitor of MNK1/2 (39), decreases PD-L1 expression on DCs without significantly affecting TAMs in mouse models of melanoma (31). The authors further demonstrate that PD-L1**^+^** DCs are likely responsible for T cell suppression (31). However, we have found that targeting MNKs using 2 distinct MNK inhibitors did not affect PD-L1 expression on tumor cells or immune cells. While we did not profile DCs in our models, we confirmed that TAMs play an active role in suppressing T cell proliferation and activity in our model. As a proof of concept, depletion of TAMs using an anti–CSF-1R antibody significantly reduced TAMs and sensitized tumors to treatment with MNK inhibitors. Interestingly, DCs and TAMs are derived from the same monocyte population and can both be suppressed by anti–CSF-1R treatment to restore T cell activity in melanomas (40). In future studies, we will evaluate the crosstalk between DCs and TAMs in mouse models of pancreatic cancer and the extent to which targeting DCs resembles the efficacy of anti–CSF-1R treatment in vivo.

Our study has some limitations. Instead of transgenic mouse models, we used syngeneic models that may not fully recapitulate the human disease. The syngeneic tumors may also not display the same fibroinflammatory reaction and the macrophage-driven TME as seen in transgenic models (37). However, our syngeneic models retained several key features of human PDAC tumors, including the lack of infiltrating CD8**^+^** T cells and the lack of response to anti–PD-1 monotherapy. Additionally, a limitation of our ex vivo slice cultures is that we could only evaluate the effects of MNK inhibitors on the immune cells already present in the tumors (37). The relative absence of CD8**^+^** T cells in human PDAC tumors precluded our ability to assess the effects of cotreatment with MNK and PD-1 inhibitors in our slice cultures studies. However, given the abundance of TAMs in human PDAC tumors, we were able to evaluate the effects of MNK inhibitors on the polarization of TAMs. Importantly, our observations in the human PDAC slice cultures support our findings in our mouse studies that MNK inhibitors polarized TAMs toward an M2 phenotype.

In summary, our results highlight the efficacy of MNK inhibitors to increase CD8**^+^** T cell infiltration and to enhance the antitumor effects of anti–PD-1 antibody in mouse models of pancreatic and differentiated thyroid cancer. Moreover, our findings also reveal an unwanted consequence of MNK inhibitors by promoting an M2 phenotype in TAMs. While inhibitors targeting MNKs (eFT508, Tomivosertib) and CSF-1R (Cabiralizumab and JNJ-40346527) are currently in clinical trials, anti–PD-1 therapies (e.g., Nivolumab, Pembrolizumab) have already been approved by the FDA for the treatment of multiple malignancies. Given the favorable safety profiles of these drugs, our study provides a rationale for pursuing combination therapies with MNK inhibitors in advanced cancer patients to enhance antitumor immune responses.

## Methods

### Cell culture.

The KPC-344 cell line, derived from a PDAC tumor developing in the *LSL-Kras^G12D/+^ × LSL-Trp53^R172H/+^* × *Pdx-1-Cre* (KPC) mouse model in the C57BL/6J background, was obtained from Sam Grimaldo (University of Illinois) and cultured in DMEM (41). The mouse thyroid cancer cell line TBP-3868, derived from a thyroid tumor developing in the *TPOCre^ER^* × *Braf^tm1Mmcm/WT^ × Trp53^tm1Brn/tm1Br^* mouse model in the B6129F1/J background, was obtained from the Parangi Lab (Massachusetts General Hospital, Boston, Massachusetts, USA) and cultured as previously described (42). The human thyroid cancer cell line MDA-T85 was obtained from MD Anderson Cancer Center (Mansfield, Ohio, USA) and cultured as previously described (43). CD18/HPAF-II and L929 cells were obtained from American Type Culture Collection (ATCC), and 8505c cells were obtained from Sigma-Aldrich. All cell lines were cultured as previously described and according to the manufacturer’s protocol. All media contained 10% FBS (Thermo Fisher Scientific) and antibiotics (100 U/mL penicillin and 100 μg/mL streptomycin; Corning).

### Chemicals and antibodies for animal studies.

CGP57380 and eFT508 were purchased from MedChemExpress (Monmouth Junction) and dissolved in 10% β-cyclodextrin (Sigma-Aldrich) for in vivo administration. Anti–PD-1 (clone RMP1-14), anti–CSF-1R (clone AFS98), and anti-CD8 (clone 2.43) mouse antibodies and their isotype-matched IgG control antibodies were purchased from Bio X Cell. Antibodies were stored undiluted at 4°C and upon treating animals were diluted in InVioPure pH 7.0 Dilution Buffer from Bio X Cell.

### Animal studies.

A 100 μL suspension of KPC-344 (2 × 10**^5^** cells/site) and TBP-3868 (1.0 × 10**^6^** cells/site) cancer cells were injected s.c. into the flanks of 6- to 8-week-old C57BL/6J and B6129SF1/J mice, respectively. After about 10–14 days when tumors achieved an approximate volume of about 70–150 mm^3^, mice were randomized and treated with the following agents: control (DMSO), CGP57380 (25 mg/kg, daily), eFT508 (1 mg/kg, daily), anti–PD-1 antibody (200 μg, twice per week), or control isotype–matched IgG antibody (200 μg, twice per week). For the macrophage-depleting experiment, an anti–CSF-1R antibody (clone AFS98, 300 μg) was administered on day –3, day 0, and subsequently in an every-other-day pattern. For the CD8-depleting experiment, an anti-CD8 antibody (clone 2.43, 200 μg) was administered on day –2, day 0, and then twice per week. Tumor volume was calculated using the formula *V* = (*W**^2^*** × *L*)/2, where *V* is tumor volume, *W* is tumor width, and *L* is tumor length by caliper measurement. Endpoint criteria included tumor volume exceeding 500 mm^3^, severe cachexia, weight loss of more than 20% body weight, and weakness or inactivity. Mice were euthanized by CO_2_ inhalation and cervical dislocation, and the tumors were excised and processed for downstream applications.

### Preparation of BMDMs.

Pelvic and femoral bones were collected from 8- to 10-week-old mice and sterilized in 70% EtOH. The bone ends were cut, and the BM was flushed out using PBS. Following treatment with blood cell lysis buffer (BioLegend), the cell pellet was resuspended and cultured for 5 days in L929-conditioned media, with media changed every 2 days. After 5 days, BMDMs were polarized toward M1 phenotype by IFN-γ (200 ng/mL) (BioLegend) treatment or M2 phenotype by IL-4 (25 ng/mL) (PeproTech) treatment for 24 hours.

### Isolation and culture of splenic T cells.

Spleens, collected from 8- to 12-week-old mice under aseptic conditions, were mechanistically disrupted through a 70 μm cell strainer into a single-cell suspension. Following treatment with blood cell lysis buffer (BioLegend), CD8**^+^** and CD4**^+^** T cells were isolated using magnetic cell sorting by negative selection (Pan T cell Isolation Kit II, Miltenyi Biotec) according to the manufacturer’s instruction. T cells were then plated in plates coated with anti-CD3 (0.5 μg/mL, clone 2C11, Bio X Cell) and anti-CD28 (5 μg/mL, clone 37.51, Bio X Cell) antibodies in RPMI 1640 medium supplemented with 10% FBS, L-glutamine (2 mM), and 2-mercaptoethanol (50 μM). After 24 hours, IL-2 (50 U/mL, PeproTech) was added to the medium. T cells were then allowed to proliferate for an additional 48 hours before treatment with MNK inhibitors. After 48 hours, T cells were collected and analyzed for the expression of CD69, PD-1, and Prf1 by flow cytometry.

### In vitro T cell proliferation assay.

Following tumor digestion, mouse TAMs (CD11b**^+^**F4/80**^+^**) were isolated and purified using the MagniSort Mouse F4/80 Positive Selection kit (Thermo Fisher Scientific). Purified T cells, isolated as described above, were stained with CellTrace CFSE (Thermo Fisher Scientific) following the manufacturer’s instruction. The labeled T cells were then plated in a 96-well plate at a density of 0.5 × 10**^5^** cells and stimulated with Dynabeads Mouse T-Activator CD3/CD28 (Thermo Fisher Scientific) in the presence of IL-2 (50 U/mL, PeproTech). TAMs were added at the indicated ratios. After incubation for 72–96 hours, T cells were harvested, stained with an anti-CD8 antibody, and analyzed by flow cytometry. The percentage and number of proliferating cells was calculated using FlowJo.

### TCGA data mining.

To evaluate the relative expression of *MKNK2, CD163*, and *MRC1* (*CD206*) in TCGA studies, their transcript abundance from RNA-Seq data, quantified as RNA-Seq by expectation maximization (RSEM), was downloaded from cBioPortal. To evaluate the relationship between *MKNK2*, *CD163*, and *MRC1* (*CD206*), correlation analysis was performed in GraphPad Prism. *P* < 0.05 was considered significant.

### Immunoblotting.

Whole-cell and whole tumor extracts were prepared in radioimmunoprecipitation (RIPA) lysis buffer supplemented with phosphatase and protease inhibitors (Calbiochem). Protein concentration was determined by the bicinchoninic acid assay (BCA) (Thermo Fisher Scientific) and separated by the SDS-PAGE. The following antibodies were used at the dilution recommended by the manufacturers: p-eIF4E (catalog 9741) and MNK1 (catalog 2195) (Cell Signaling Technology), eIF4E (catalog sc-9976) and HSP90 (catalog sc-7940) (Santa Cruz Biotechnology), and mouse PD-L1 (catalog AF1019) (R&D Systems). Secondary anti–mouse IgG (catalog A4416) and anti–rabbit IgG (catalog A6667) antibodies were purchased from Sigma-Aldrich and used a 1:4000 dilution. Images of blots were acquired on HyBlot ES Autoradiography Film (Thomas Scientific) following incubation with SuperSignal West Pico PLUS (Thermo Fisher Scientific).

### Membrane-based cytokine array.

The membrane-based immunoassay was purchased from R&D Systems (catalogs ARY006 and ARY028). Whole-cell extracts were collected from at least 3 individual tumors, incubated with the membranes overnight, and they were analyzed for the expression of different apoptotic proteins following the manufacturer’s instructions. Each assay was repeated at least twice. Pixel density was quantified by ImageJ (NIH).

### IHC staining.

Human thyroid tissue microarrays (TMAs) were purchased from US Biomax, and human PDAC TMAs were created by the Pathology Core Facility at Northwestern University from de-identified PDAC specimens. Human samples were stained for the following antibodies from Abcam: p-eIF4E (catalog ab76256, 1:2000), CD8 (catalog ab4055, 1:10000), Arginase-1 (catalog ab133543, 1:2000), CD163 (catalog ab156769, 1:1000), CD206 (catalog ab64693, 1:1000), and MHCII (catalog ab55152, 5 μg/mL). CD80 (catalog MAB140, 5 μg/mL) was purchased from R&D Systems, CD86 (catalog SPM600, 4 μg/mL) was purchased from Novus Biologicals, and CD68 catalog 76437, 1:500) was purchased from Cell Signaling Technology. Mouse tumors were stained for CD8 (catalog 98941, 1:1000), Ki67 (catalog 12202, 1:1000), and F4/80 (catalog 70076, 1:1000) using antibodies obtained from Cell Signaling Technology and with GzmB (catalog ab255598, 1:1000) and Arginase-1 (catalog ab203490, 1:1000) using antibodies purchased from Abcam. Antigen retrieval was carried out as previously described (26, 32) and according to the manufacturers’ instructions. After antigen retrieval, tumor/tissue sections were incubated with BLOXALL (Vector Laboratories) for 15 minutes and then incubated with the Fc receptor blocker (Innovex) for 20 minutes at room temperature. Sections were incubated with primary antibodies at the recommended dilutions in 1% BSA/PBS overnight at 4°C. ImmPRESS Secondary HRP anti–rabbit IgG and anti–mouse IgG (Peroxidase) Polymer Detection Kit was purchased from Vector Laboratories. Photographs were taken on the FeinOptic microscope and the Jenoptik ProgRes C5 camera and analyzed by ImageJ. Whole tumor expression of CD8, p-eIF4E, F4/80, and Arginase-1 levels was obtained by TissueGnostics and analyzed by HistoQuest.

### Immunofluorescence staining.

Antigen retrieval was carried out as previously described using either pH 6.0 or pH 9.0 buffer (26, 32). After antigen retrieval, tumor/tissue sections were incubated with 10% normal goat serum (Agilent) for 20 minutes at room temperature, followed by incubation with 1% BSA/PBS buffer for 1 hour at room temperature. The tissues were then incubated with primary antibodies in 1% BSA/PBS overnight at 4°C. The following primary antibodies were used: p-eIF4E (Abcam, catalog ab76256, 1:2000), Ki67 (Abcam, catalog ab15580, 1:1000), and cytokeratin 8 (DSHB, TROMA-I, 5 μg/mL) in 1% BSA/PBS overnight at 4°C. Secondary Alexa Fluor 488 and Alexa Fluor 594 antibodies were purchased from Thermo Fisher Scientific and used at 1:1000 dilution factor. DAPI was used to counterstain the nuclei. Final pictures were taken on an EVOS M5000 microscope.

### Flow cytometry staining and analysis.

Mouse tumors were mechanically disrupted and passed through a 70 μm cell strainer in PBS buffer supplemented with 5% FBS. Cell pellets were pretreated with blood cell lysis buffer (BioLegend) and then incubated with TruStain FcX (1:100) (BioLegend) for 20 minutes on ice. All subsequent primary antibodies were stained for 45 minutes on ice using antibodies purchased from BioLegend: CD45-FITC (clone 30-F11), CD45-BV570 (clone 30-F11), CD45-BV711 (clone 30F-11), CD3-FITC (clone 17A2), CD4–APC-Cy7 (clone RM4-5), CD8–PerCP-Cy5.5 (clone 53-6.7), CD8-AF594 (clone 53-6.7), PD-1–PE (clone 29F.1A12), PD-1–APC (clone RMP1-30), PD-1–BV605 (clone 29F.1A12), CD69-APC (clone H1.2F3), CD69-PE (clone H1.2F3), F4/80-PE (clone BM8), CD11b-APC (clone M1/70), CD11b-FITC (clone M1/70), Ly6G–PE-Cy7 (clone 1A8), Ly6C-AF700 (clone HK1.4), PD-L1–APC (clone 10F.9G2), PD-L1–BV605 (clone 10F.9G2), CD19-AF594 (clone 6D5), NK1.1–PE-Cy5 (clone PK136), FoxP3-PE (clone MF-14), TNF-α–AF647 (clone MP6-XT22), GzmB-FITC (clone GB11), and Prf1-PE (clone S16009B). PD-L1–PE (clone MIH5), Prf1-APC (clone 17-9392-80), Arginase-1–APC (clone A1exF5), and Arginase-1–PE-Cy7 (clone A1exF5) were purchased from Thermo Fisher Scientific. In addition to the primary antibodies, all samples were incubated with LIVE/DEAD fixable blue dead cell stain (Thermo Fisher Scientific, 1:1000) to determine viability. For intracellular cytokine staining, the samples were preincubated with GolgiStop (1:1000) (BD Biosciences) and permeabilized by the intracellular permeabilization wash buffer (BioLegend) following the manufacturer’s instructions. After treatment with MNK inhibitors or MNK1/2 siRNAs for the indicated times, human and mouse cancer cells were stained for PD-L1 (clone MIH5, PE, Thermo Fisher Scientific or clone 10F.9G2, APC, BioLegend) following the same protocol. Similarly, ex vivo expanded splenic T cells were stained for CD8, PD-1, CD69, Prf1, GzmB, and TNF-α. Samples were acquired on a 3C.A1 LSR Fortessa 1 Analyzer or a 4C.A2 BD FACSymphony A5-Laser Analyzer. Obtained data were analyzed by FlowJo, and subsequent analysis was performed using GraphPad. The samples were gated as detailed in Supplemental Figures 10–14.

### Slice culture conditions and treatments.

Following the previously published protocol (38), human PDAC tumors were sectioned into 250 μm–thick slices using the Leica VT1000S Vibrating blade microtome. The slices were then placed atop collagen-coated 0.4 μm pore membrane inserts in 6-well plates. The next day, the slice cultures were treated with different drugs at the indicated doses. Fresh treatment media was replaced every 2–3 days. At the end of the experiment, slices were fixed with 4% PFA and processed by the Pathology Core Facility at Northwestern University. The embedded tissues were subsequently embedded and sectioned for staining following the protocols described above.

### Transfection.

The siRNAs targeting mouse-specific *Mknk1* (Mnk1) and *Mknk2* (Mnk2) were obtained from Ambion Inc. (Thermo Fisher Scientific). All siRNA transfections were carried out using RNAimax reagent (Thermo Fisher Scientific) according to the manufacturer’s instructions. The following siRNA sequences were used: mouse *Mknk1* (sense: GCACUUCAAUGAGCGAGAAtt and antisense: UUCUCGCUCAUUGAAGUGCtt), mouse *Mknk2* (sense: GGGACAUAGGAAUGUUCUAtt and antisense: UAGAACAUUCCUAUGUCCCtg). Transfected cells were collected after 48 hours, and the expression levels of target genes were evaluated by quantitative PCR (qPCR) and/or Western blotting.

### qPCR analysis.

Quantitative gene expression was performed with gene-specific TaqMan probes, TaqMan Universal PCR Master Mix, and the CFX Connect Real-Time PCR System from Bio-Rad. The data were then quantified with the comparative Ct method for relative gene expression. GAPDH was used as the housekeeping control.

### Statistics.

Data are represented as mean ± SEM or as mean ± SD, as specified in figure legends. The values for *n*, *P*, and the specific statistical test performed for each experiment (2-tailed unpaired *t* test, 2-tailed paired *t* test, 1-way ANOVA, 2-way ANOVA, log-ranked [Mantel-Cox] test for survival analysis, and correlation analyses) are indicated in figure legends. All statistical analyses were done using GraphPad Instat. *P* < 0.05 was considered significant.

### Study approval.

All animal work and procedures were approved by the Northwestern University IACUC. In addition, all animal experiments were performed in accordance with relevant guidelines and regulations. For human studies, pancreatic tissue was obtained from patients with PDAC undergoing resection on a protocol approved by the IRB of Northwestern University. Informed consent was obtained from patients before resection. The resected specimens, which were processed for histology and IHC studies and ex vivo cultures, were deidentified.

## Author contributions

TNDP designed the studies, performed the experiments, analyzed the data, and wrote the manuscript. CS, MAS, AEM, DNS, and MGK performed the experiments. DRP assisted in the design of flow cytometry experiments. DJB provided human PDAC samples and assisted in the ex vivo human slice culture experiments. HGM designed the studies, analyzed the data, wrote and edited the manuscript, and secured funding. All authors edited and approved the final manuscript.

## Supplementary Material

Supplemental data

## Version 1 04/05/2022

In-Press Preview

## Version 2 05/09/2022

Electronic publication

## Version 3 05/24/2022

Slight change in title
