# Supplementary material for: Inhibition of MNKs promotes macrophage immunosuppressive phenotype to limit CD8+ T cell antitumor immunity
Source: JCI Insight. 2022 May 9;7(9):e152731. doi: 10.1172/jci.insight.152731 (PMC9090262; doi:10.1172/jci.insight.152731)
Supplement: Supplemental data [file jciinsight-7-152731-s010.pdf]

Inhibiting MNK kinases promotes macrophage immunosuppressive phenotype to limit CD8$^+$ T cell anti-tumor immunity

Thao N.D. Pham[1,2,*], Christina Spaulding[1,2], Mario A. Shields[1,3], Anastasia E. Metropulos[1], Dhavan N. Shah[4], Mahmoud G. Khalafalla[1], Daniel R. Principe[5], David J. Bentrem[2,3,4], Hidayatullah G. Munshi[1,2,3,*]

[1]Department of Medicine, Feinberg School of Medicine, Northwestern University, Chicago, IL, USA
[2]Jesse Brown VA Medical Center, Chicago, IL, USA
[3]The Robert H. Lurie Comprehensive Cancer Center, Chicago, IL, USA
[4]Department of Surgery, Feinberg School of Medicine, Northwestern University, Chicago, IL, USA
[5]Medical Scientist Training Program, College of Medicine, University of Illinois at Chicago, Chicago, IL, USA

***Corresponding authors:** Thao N. D. Pham, Ph.D. (thao.pham@northwestern.edu) or Hidayatullah G. Munshi, M.D. (h-munshi@northwestern.edu)

Supplemental Figures and Figure Legends

Supplemental Fig. S1

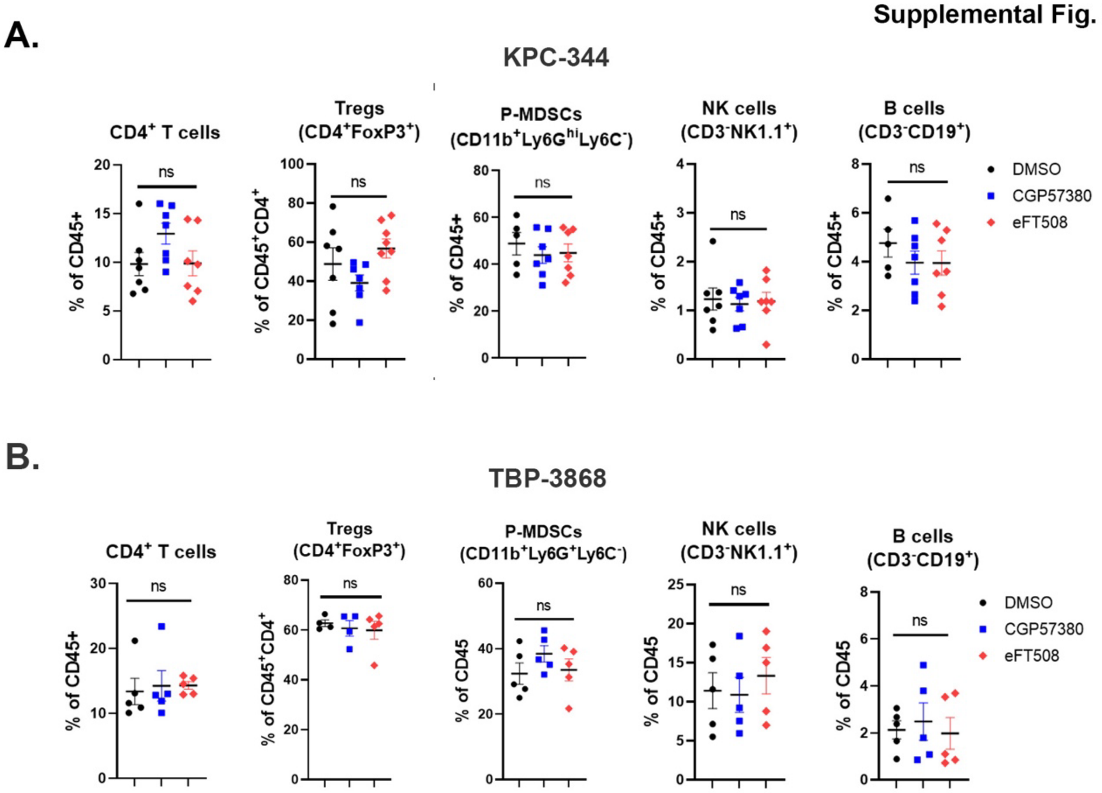

Supplemental Fig. S1. MNK inhibitors do not affect major immune cell populations. (**A** and **B**) Mice with established syngeneic pancreatic (KPC-344) and thyroid (TBP-3868) tumors were randomized and treated with DMSO (vehicle control), CGP57380 (25 mg/kg), or eFT508 (1mg/kg) daily for 2 weeks. At the study endpoint, tumors were collected, digested, and analyzed by flow cytometry for the frequency of $CD4^+$ T cells ($CD45^+CD4^+$), regulatory T cells (Tregs, $CD45^+CD4^+FoxP3^+$), polymorphonuclear myeloid-derived suppressive cells (P-MDSCs, $CD45^+CD11b^+Ly6G^{hi}Ly6C^-$), NK cells ($CD45^+CD3^-NK1.1^+$), and B cells ($CD45^+CD3^-CD19^+$) as described in Materials and Methods. Data points represent individual tumors. Error bars represent SEM, and analysis was done using one-way ANOVA followed by Dunnett's multiple comparison test. ns, non-significant.

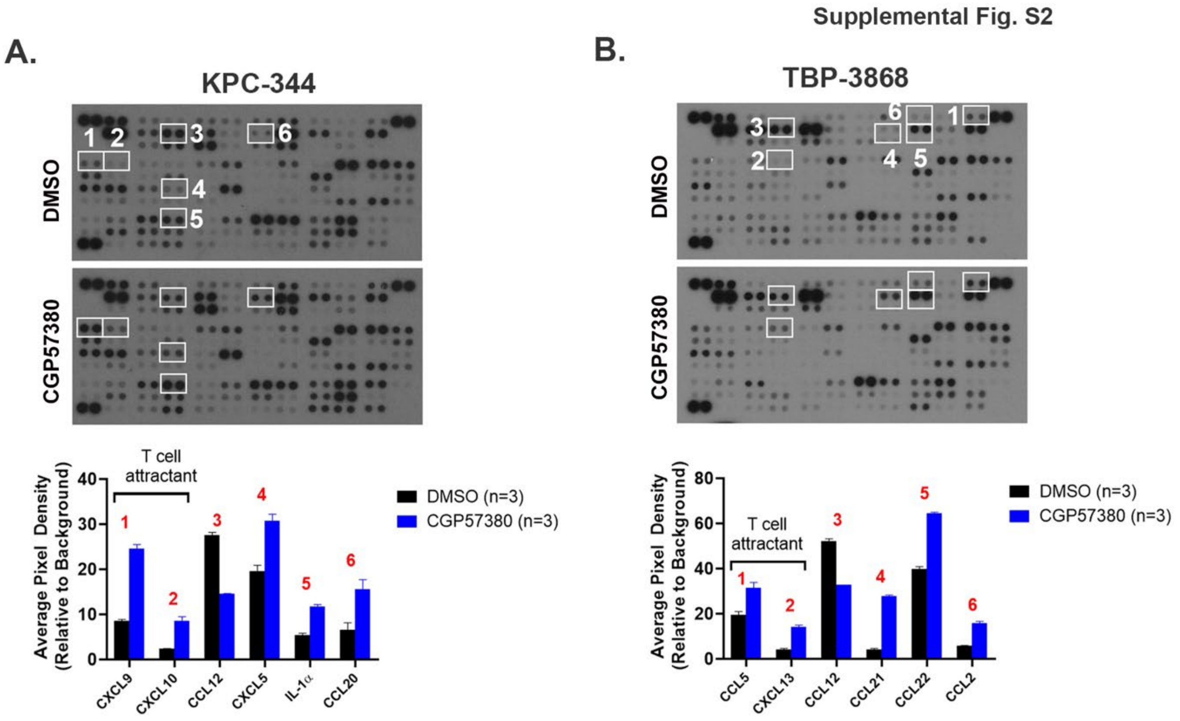

Supplemental Fig. S2: MNK inhibitors increase T cell chemo-attractants. (**A** and **B**) Mice with established syngeneic pancreatic (KPC-344) and thyroid (TBP-3868) tumors were randomized and treated with DMSO (vehicle control) or CGP57380 (25 mg/kg) daily for 2 weeks. At the study endpoint, tumors were collected and subjected to cytokine analysis per the manufacturer's instructions. Pixel intensity was analyzed by ImageJ. Three individual tumors from each treatment group were pooled for the analysis. Error bars represent SD from two technical replicates. Data are representative of two biological experiments.

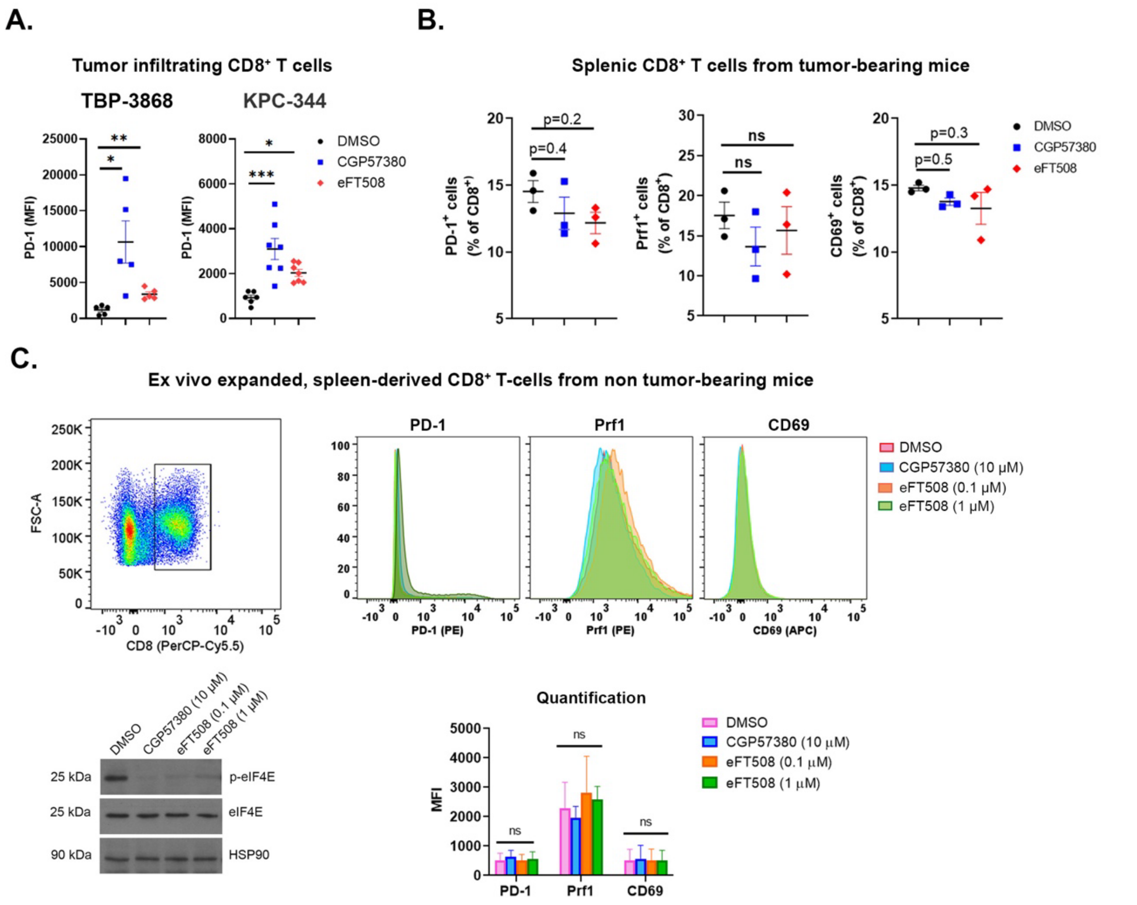

Supplemental Fig. S3: MNK inhibitors do not induce exhaustion in non-tumor-infiltrating CD8$^+$ T cells. (**A**) Mice with established syngeneic pancreatic (KPC-344) and thyroid (TBP-3868) tumors were randomized and treated with DMSO (vehicle control), CGP57380 (25 mg/kg), or eFT508 (1mg/kg) daily for 2 weeks. At the study endpoint, the tumor-infiltrating CD8$^+$ T cells were analyzed by flow cytometry for PD-1, and the mean fluorescence intensity (MFI) was calculated. (**B**) Mice with established syngeneic pancreatic (KPC-344) tumors were randomized and treated with DMSO (vehicle control), CGP57380 (25 mg/kg), or eFT508 (1mg/kg) daily for 2 weeks. At the study endpoint, CD8$^+$ T cells were isolated from the spleen of the tumor-bearing mice and evaluated by flow cytometry for PD-1, Perforin-1 (Prf1), and CD69 expression. (**C**) CD8$^+$ T cells were isolated from the spleen of non-tumor-bearing mice, expanded *ex vivo* by CD3 (0.5 μg/mL) and CD28 (5 μg/mL) co-ligation and stimulated by IL-2 (50 U/mL) as described in Materials and

Methods. The expanded CD8⁺ T cells were then treated with CGP57380 (10 μM) or eFT508 (0.1, 1 μM) for 48 hours. The activity of MNK inhibitors was confirmed by western blotting for eIF4E phosphorylation using HSP90 as a loading control. Blot is representative of three biological replicates. The resulting CD8⁺ T cells were analyzed by flow cytometry for PD-1, Prf1, and CD69 expression. Data points in **A** and **B** represent individual tumors. Error bars in **A**-**C** represent SEM. Analysis was done by one-way ANOVA, followed by Dunnett's post-test. *, $p<0.05$ **, $p<0.01$ ***, $p<0.001$; ns, non-significant.

Supplemental Fig. S4

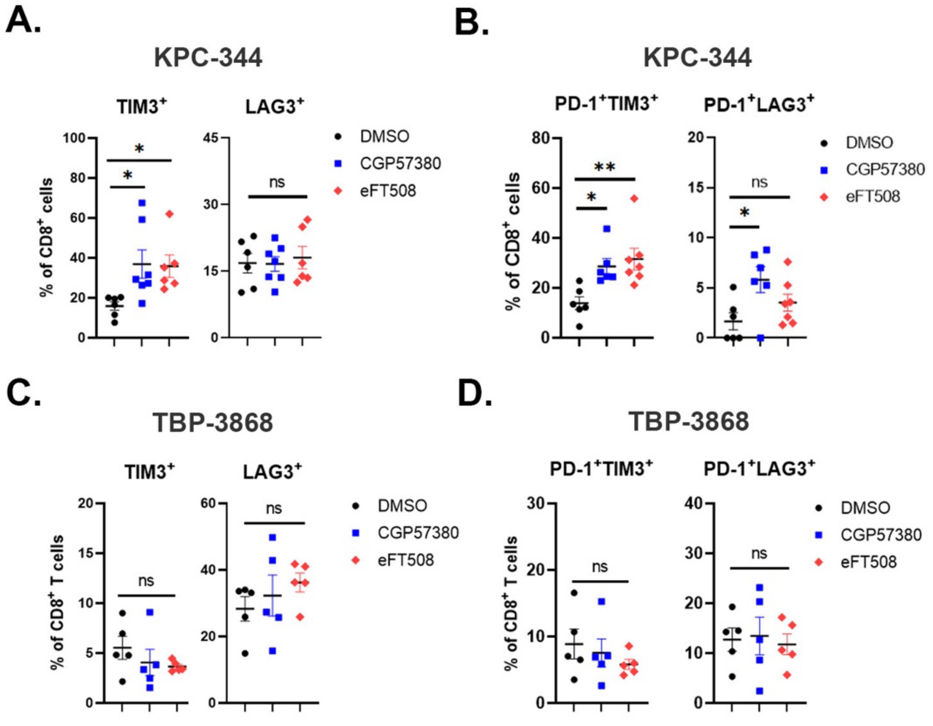

Supplemental Fig. S4: Effect of MNK inhibitors on TIM3 and LAG3 expression by CD8$^+$ T cells. (**A–D**) Mice with established syngeneic pancreatic (KPC-344) and thyroid (TBP-3868) tumors were randomized and treated with DMSO (vehicle control), CGP57380 (25 mg/kg), or eFT508 (1mg/kg) daily for 2 weeks. At the study endpoint, the tumor-infiltrating CD8$^+$ T cells were evaluated by flow cytometry for expression of TIM3 or LAG3 or co-expression of TIM3 and PD-1 or co-expression of LAG3 and PD-1. Data points represent individual tumors. Error bars represent SEM. Analysis was done by one-way ANOVA, followed by Dunnett's post-test. *, $p<0.05$ **, $p<0.01$; ns, non-significant.

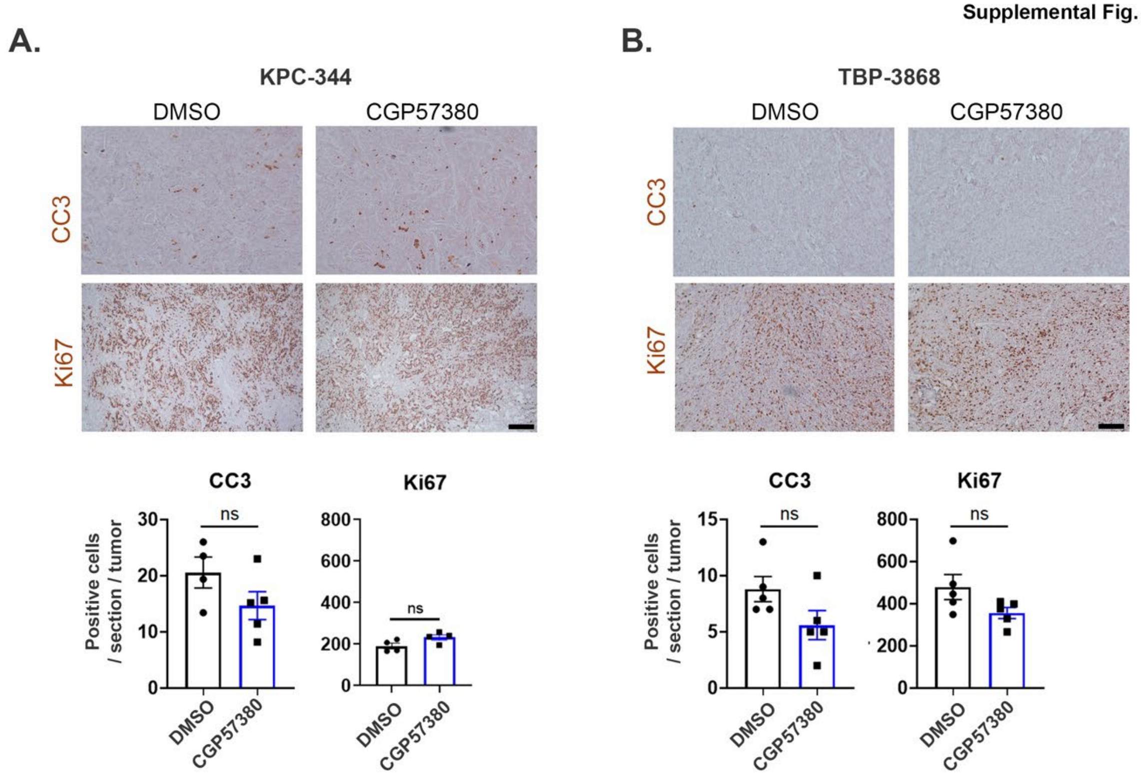

Supplemental Fig. S5: MNK inhibitors do not affect tumor proliferation or apoptosis. (**A** and **B**) Mice with established syngeneic pancreatic (KPC-344) and thyroid (TBP-3868) tumors were randomized and treated with DMSO (vehicle control) or CGP57380 (25 mg/kg) daily for 2 weeks. At the study endpoint, the tumors were collected and stained for cleaved caspase-3 (CC3) and Ki67 by immunohistochemistry. The number of positive cells from each tumor was counted and averaged from five 10X sections. Scale bar, 100 μm. Data points represent individual tumors. Error bars represent SEM, and analysis was undertaken using unpaired Student's *t* test. ns, non-significant.

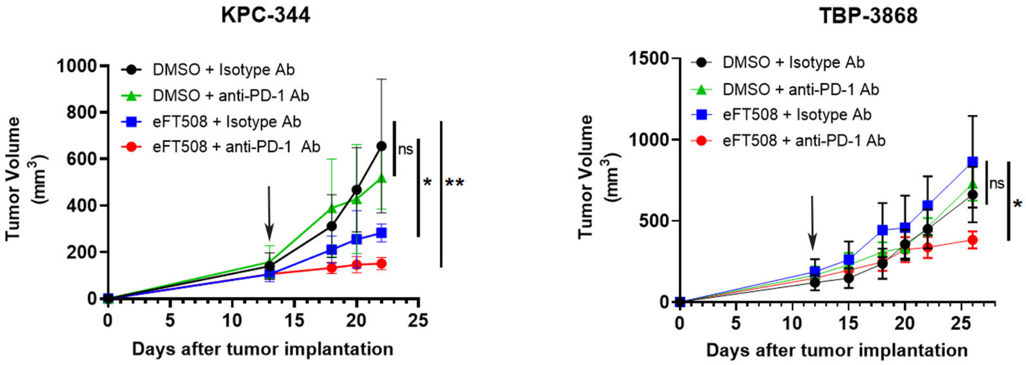

Supplemental Fig. S6: Anti-PD-1 antibody enhances the anti-tumor efficacy of the MNK inhibitor eFT508. Mice with established KPC-344 and TBP-3868 tumors were randomized and treated with eFT508 (1 mg/kg, daily) or DMSO (vehicle control) in combination with an anti-PD-1 antibody (200 µg, twice weekly) or a control isotype-matched IgG antibody (200 µg, twice weekly). Tumor size was measured daily by caliper, and the tumor volume was calculated using the formula $V = (W^2 \times L)/2$. Arrows indicate start of treatment. Error bars represent SEM. Analysis was done by one-way ANOVA, followed by Dunnett's post-test; *, $p<0.05$; **, $p<0.01$; ns, non-significant.

Supplemental Fig. S7

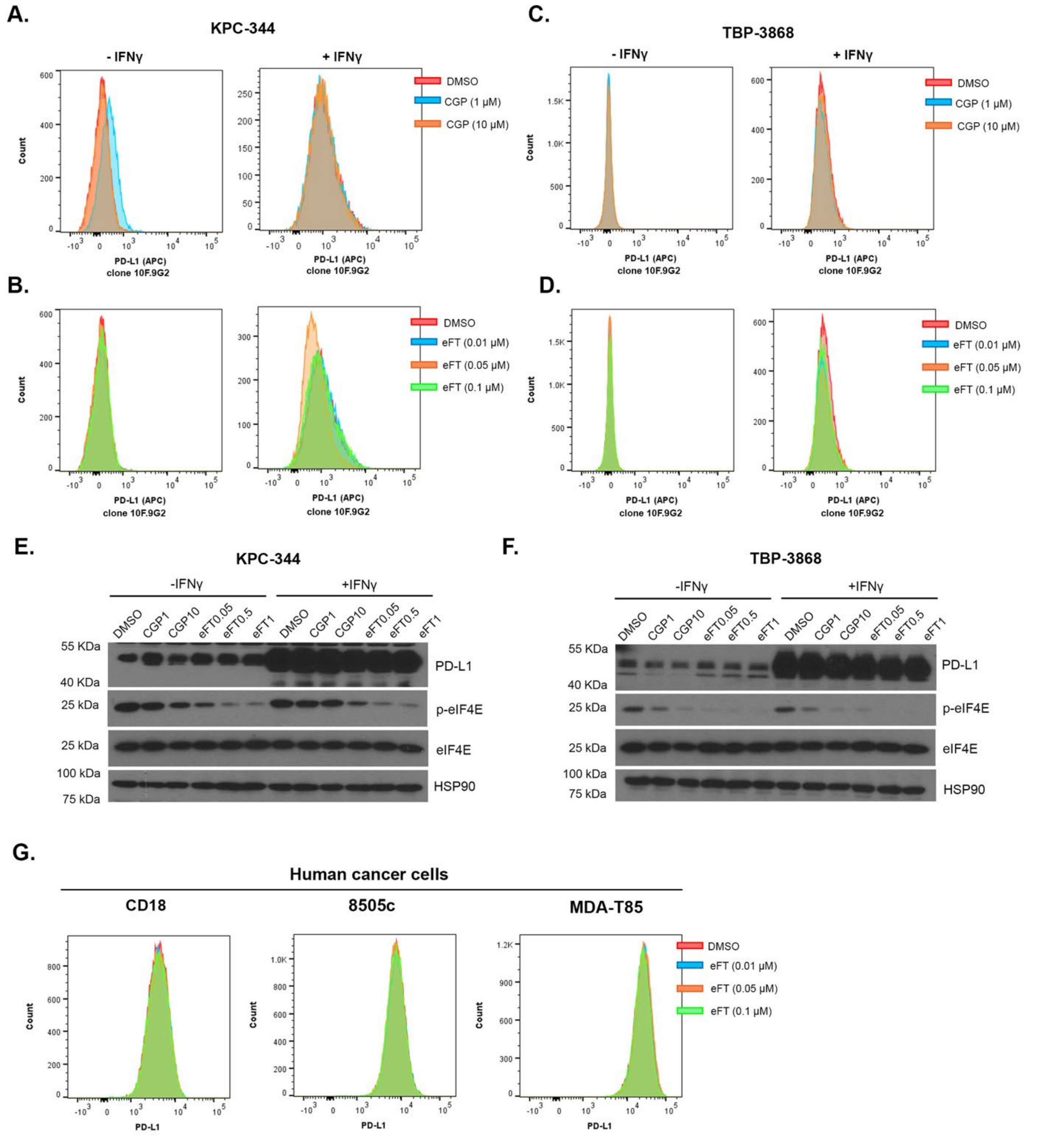

Supplemental Fig. S7: MNK inhibitors do not affect PD-L1 expression *in vitro*. (**A–D**) Mouse pancreatic KPC-344 and thyroid TBP-3868 cancer cells were pre-treated with DMSO, CGP57380 (CGP; 1, 10 μM), or eFT508 (eFT; 0.01, 0.05, 0.1 μM) for 24 hours and then treated with vehicle control or IFNγ (200 ng/mL) for additional 24 hours. The cells were then analyzed for PD-L1

expression using an APC-conjugated antibody by flow cytometry. (**E** and **F**) Mouse pancreatic KPC-344 and thyroid TBP-3868 cancer cells were pre-treated with DMSO, CGP57380 (CGP; 1, 10 μM), or eFT508 (eFT; 0.05, 0.5, 1 μM) for 24 hours and then treated with vehicle control or IFNγ (200 ng/mL) for additional 24 hours. PD-L1 expression was analyzed by western blotting. The blot here is representative of three independent experiments. (**G**) Human pancreatic (CD18) and thyroid (8505c, MDA-T85) cancer cells were pre-treated with DMSO or eFT508 (0.01, 0.05, 0.1 μM) for 24 hours and then treated with vehicle control or human IFNγ (200 ng/mL) for additional 24 hours. The cells were then analyzed for PD-L1 expression using a PE-conjugated antibody by flow cytometry.

Supplemental Fig. S8

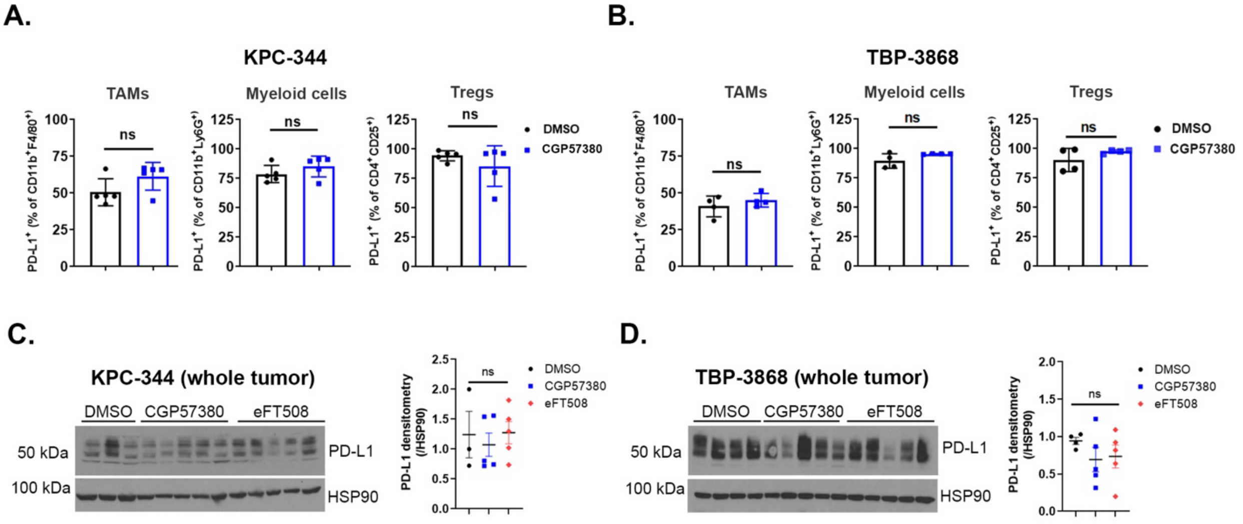

Supplemental Fig. S8: MNK inhibitors do not affect PD-L1 expression *in vivo*. (**A** and **B**) Mice with established syngeneic pancreatic (KPC-344) and thyroid (TBP-3868) tumors were randomized and treated with DMSO (vehicle control) or CGP57380 (25 mg/kg) daily for 2 weeks. At the study endpoint, the tumor-associated macrophages (TAMs, $CD11b^{+}F4/80^{+}$), myeloid cells ($CD11b^{+}Ly6G^{+}$), and regulatory T cells (Tregs, $CD4^{+}CD25^{+}$) were isolated and analyzed for PD-L1 expression by flow cytometry and the FlowJo. Data points represent individual tumors. Error bars represent SEM, and analysis was undertaken using unpaired Student's *t* test; ns, non-significant. (**C** and **D**) Mice with established syngeneic pancreatic (KPC-344) and thyroid (TBP-3868) tumors were randomized and treated with DMSO (vehicle control), CGP57380 (25 mg/kg), or eFT508 (1mg/kg) daily for 2 weeks. At the study endpoint, PD-L1 expression in the whole tumor was analyzed by western blotting. Data points represent individual tumors. Error bars represent SEM, and analysis was done by one-way ANOVA, followed by Dunnett's post-test; ns, non-significant.

Supplemental Fig. S9

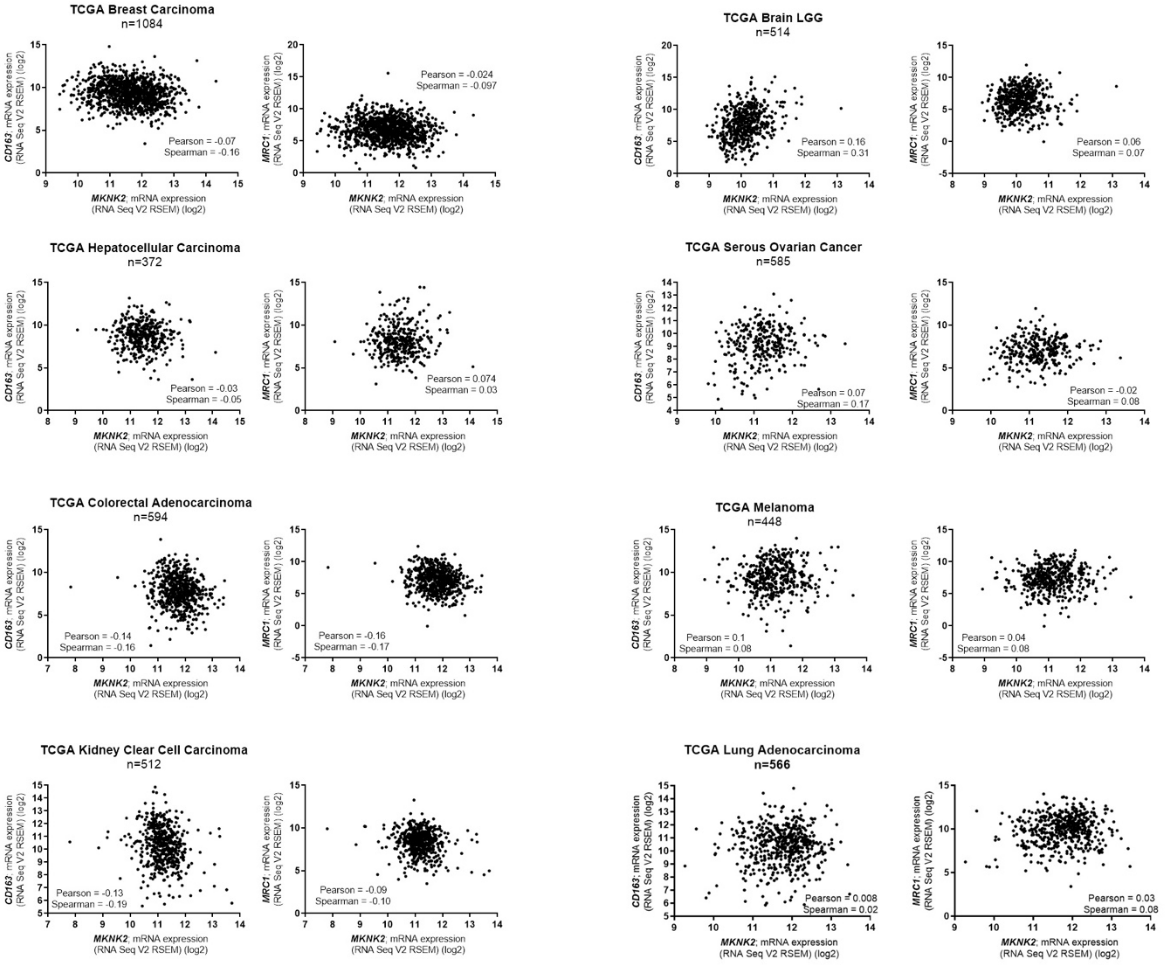

Supplemental Fig. S9: Correlation analysis of *MKNK2*, *CD163*, and *MRC1* (*CD206*) in TCGA studies. The relative expression of *MKNK2, CD163, and MRC1* (*CD206*) in TCGA studies and their transcript abundance from RNA-seq data (quantified as RSEM) were downloaded from cBioPortal. Correlation analysis was performed in GraphPad Prism to evaluate the relationship between *MKNK2*, *CD163, and MRC1* (*CD206*).

Supplemental Fig. S10

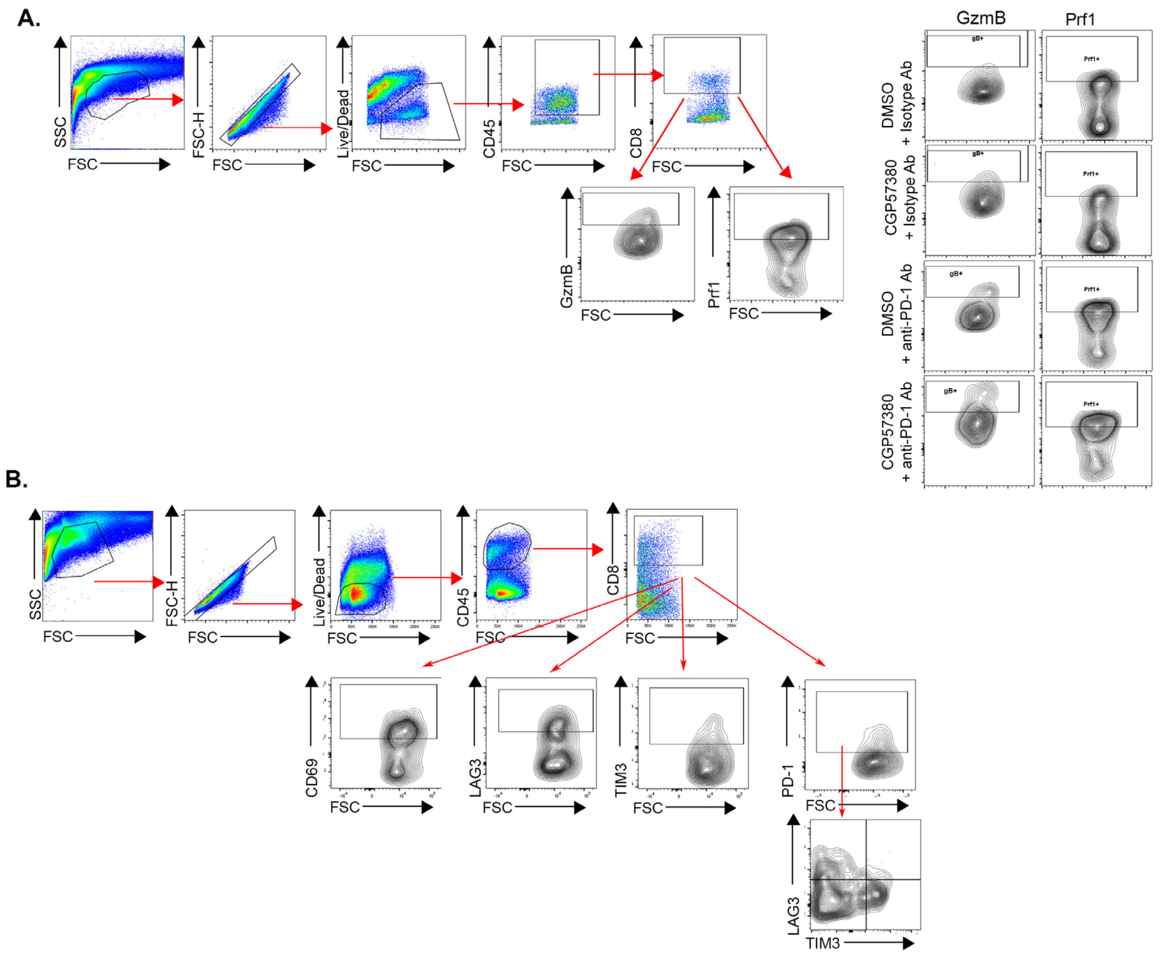

Supplemental Fig. S10: Gating strategy for cytolytic and exhaustion markers in CD8⁺ T cells. (A) Gating strategy for expression of granzyme B (Gnzb) and perforin-1 (Prf1). Representative data from tumors treated with CGP57380 or DMSO (vehicle control) in combination with either an anti-PD-1 antibody or a control isotype-matched IgG antibody. (**B**) Gating strategy for expression of CD69, LAG3, TIM3, and PD-1.

Supplemental Fig. S11

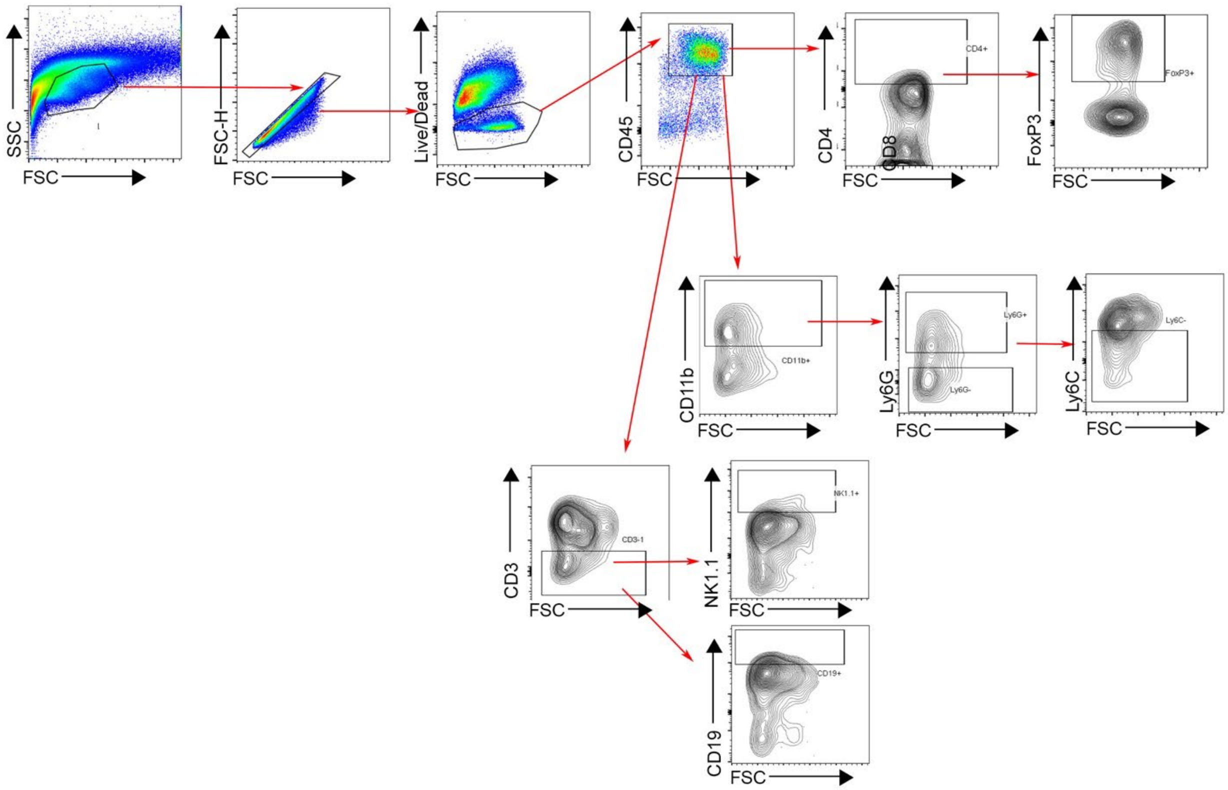

Supplemental Fig. S11: Gating strategy for CD4^{+}, regulatory T cells (Tregs), polymorphonuclear myeloid-derived suppressor cells (P-MDSCs), natural killer (NK) cells, and B cells.

Supplemental Fig. S12

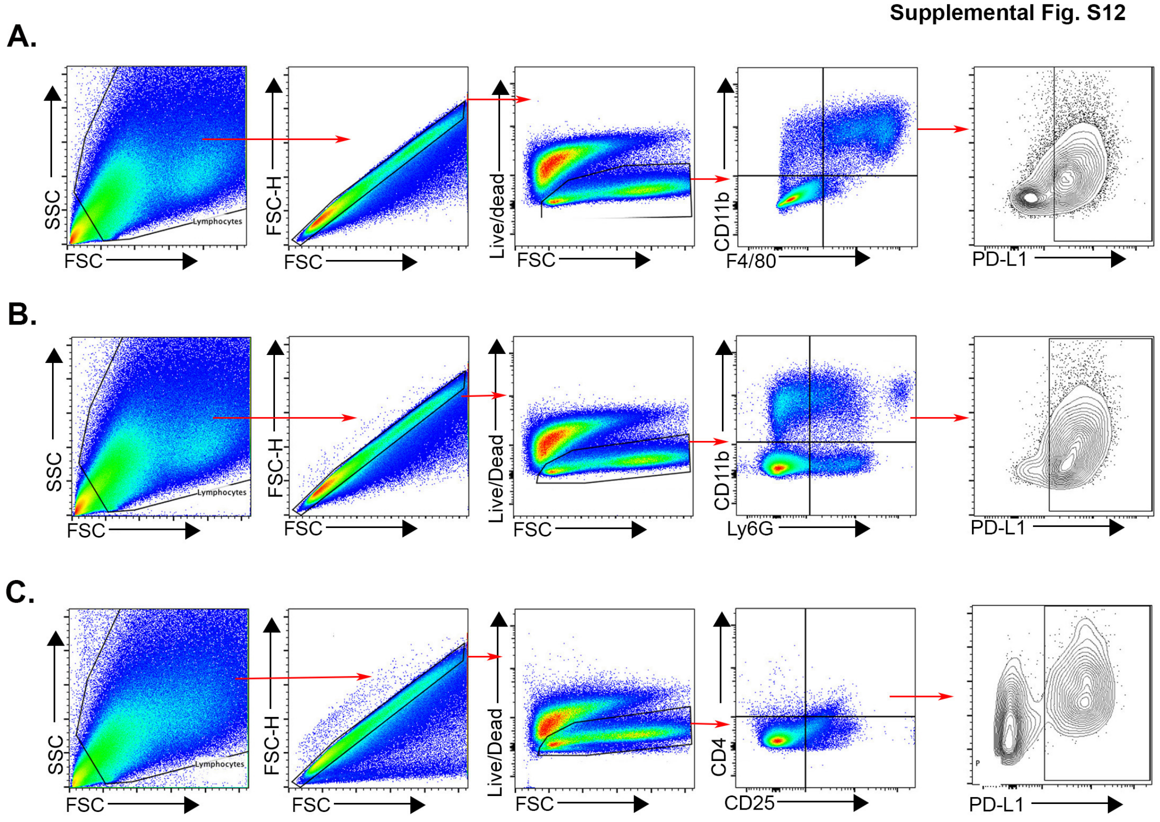

Supplemental Fig. S12: Gating strategy for the expression of PD-L1 on tumor-associated macrophages (**A**), myeloid cells (**B**), and regulatory T cells (**C**).

Supplemental Fig. S13

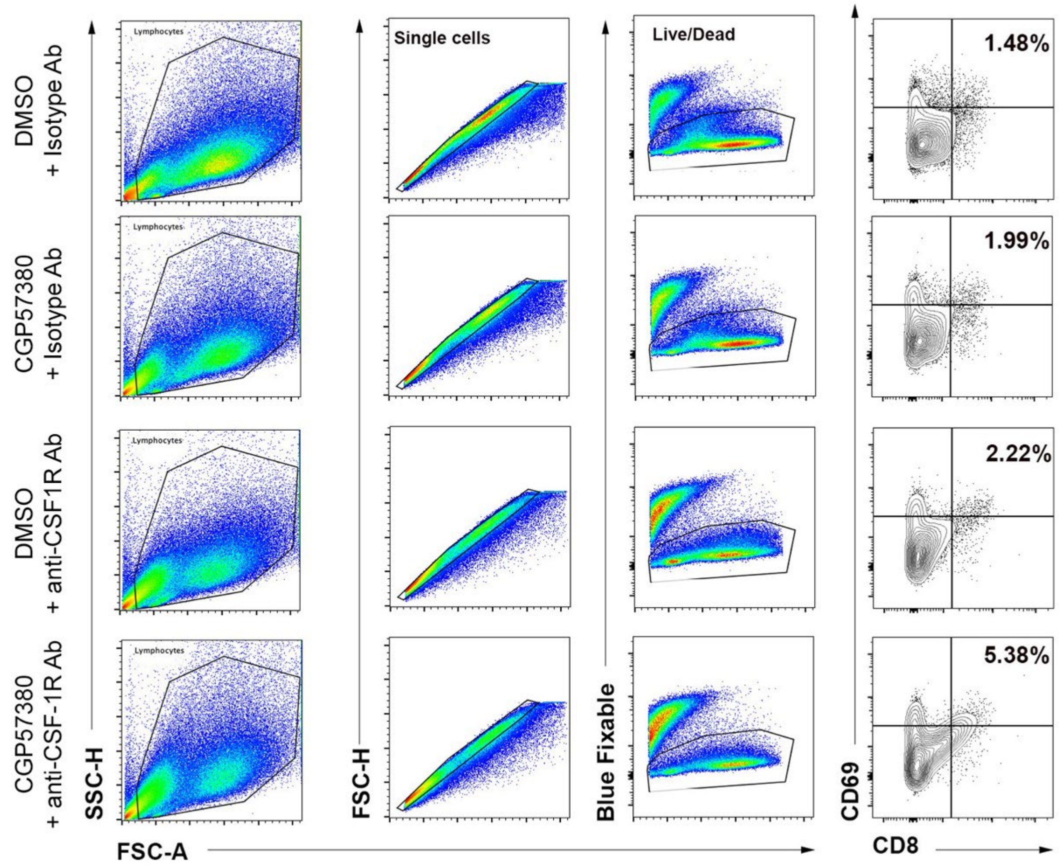

Supplemental Fig. S13: Gating strategy for CD69^{+}CD8^{+} T cells from tumors treated with CGP57380 or DMSO (vehicle control) in combination with either an anti-CSF-1R antibody or a control isotype-matched IgG antibody.

Supplemental Fig. S14

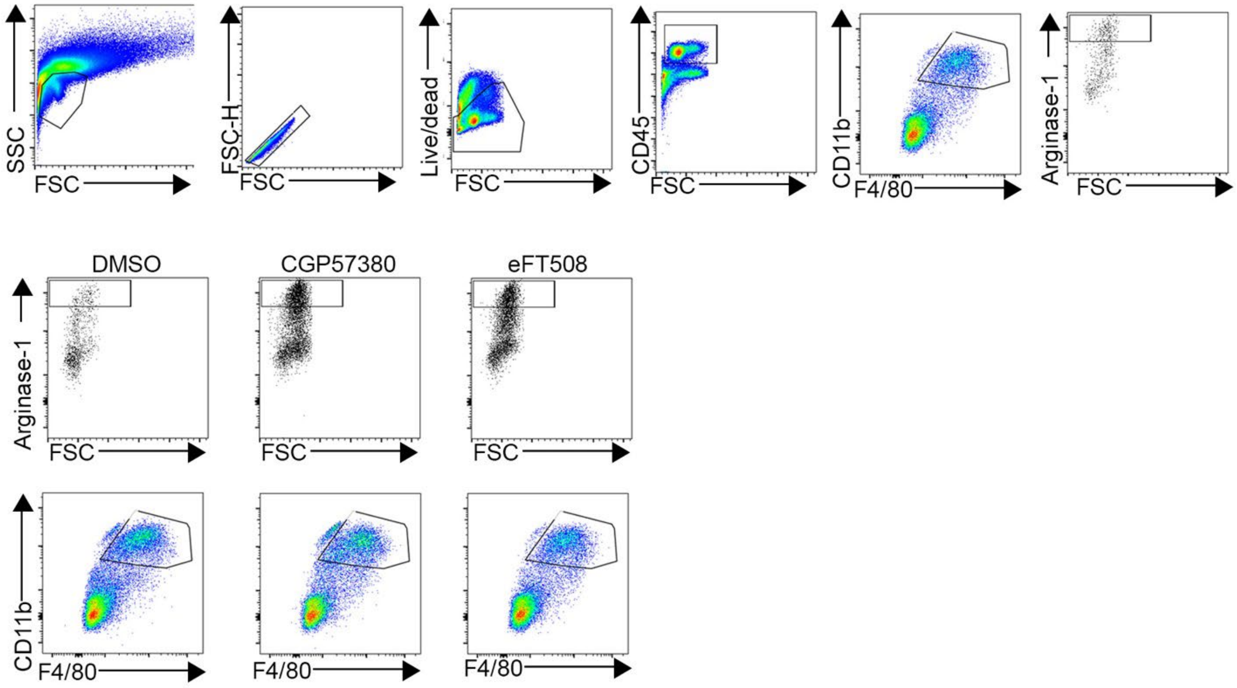

Supplemental Fig. S14: Gating strategy for Arginase-1 expression in tumor-associated macrophages. Representative data from tumors treated with DMSO, CGP57380, or eFT508
